# Direct Benzene Hydroxylation with Dioxygen Induced by Copper Complexes: Uncovering the Active Species by DFT Calculations

**DOI:** 10.1021/acs.organomet.2c00202

**Published:** 2022-07-14

**Authors:** Elena Borrego, Laura Tiessler-Sala, Jesus J. Lázaro, Ana Caballero, Pedro J. Pérez, Agustí Lledós

**Affiliations:** † Laboratorio de Catálisis Homogénea, Unidad Asociada al CSIC, CIQSO-Centro de Investigación en Química Sostenible and Departamento de Química, Universidad de Huelva, Huelva 21007, Spain; ‡ Departament de Química, Universitat Autònoma de Barcelona, Cerdanyola del Vallès, Barcelona 08193, Spain; § Cepsa Research Center, Compañía Española de Petróleos S.A., Alcalá de Henares, Madrid 28850, Spain

## Abstract

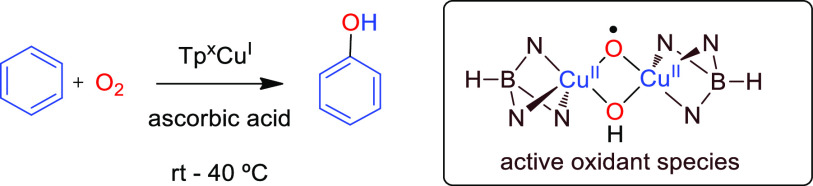

The direct oxidation of benzene into phenol using molecular oxygen at very mild temperatures can be promoted in the presence of the copper complex Tp^Br3^Cu(NCMe) in the homogeneous phase in the presence of ascorbic acid as the source of protons and electrons. The stoichiometric nature, relative to copper, of this transformation prompted a thorough DFT study in order to understand the reaction pathway. As a result, the dinuclear species Tp^Br3^Cu^II^(μ-O^•^)(μ-OH)Cu^II^Tp^Br3^ is proposed as the relevant structure which is responsible for activating the arene C–H bond leading to phenol formation.

## Introduction

**Scheme 1:**
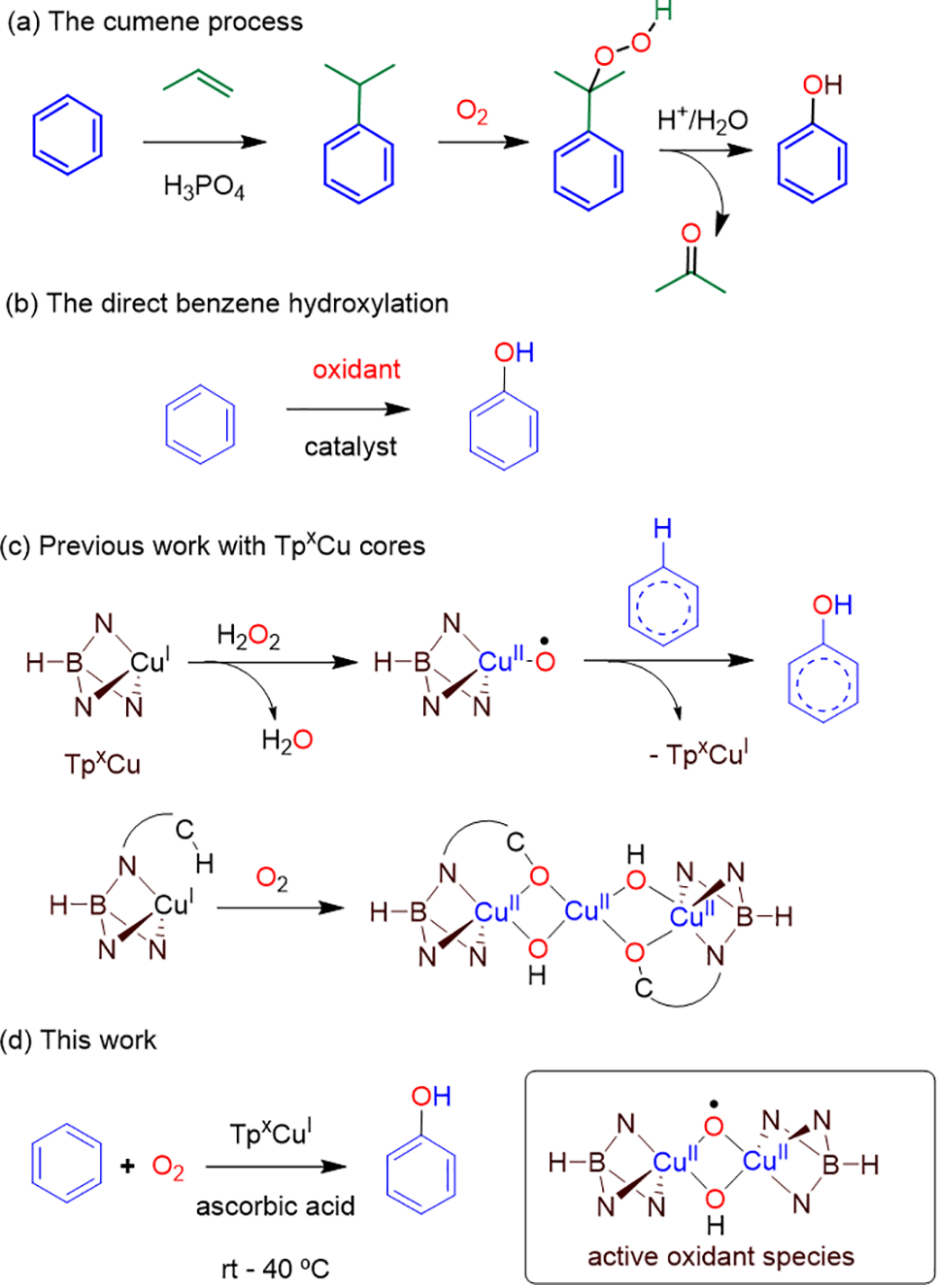
Benzene Oxidation into Phenol

One of the pillars of industrial chemistry is phenol given its wide use in the production of several chemicals at multiton scale.^1^ Despite such importance, the method employed is far of being efficient in terms of yields, which is below 10% based on benzene. This so-called cumene process consists of three steps and employs O_2_ as the oxidant (Scheme 1a).^2^ The ideal transformation would consist of the direct hydroxylation of the benzene C–H bond (Scheme 1b),^3^ which has been described with several oxidants such as H_2_O_2_, N_2_O, or O_2_. The use of molecular oxygen is a quite challenging transformation due to the bond dissociation energies of both the dioxygen molecule and the arene C–H bond.

Studies aimed at the aerobic benzene hydroxylation have mainly employed heterogeneous catalysts.^4^ In the homogeneous counterpart, examples are scarce, with a vanadium-based system dominating the area.^5^ In all cases, moderate to low yields have always been described, with a 20% yield barely surpassed. Most of these systems require the support of a reductant which is consumed in a stoichiometric manner relative to produced phenol.

Copper has been widely employed to promote oxidation in view of its role in biology through metalloenzymes.^6^ Our group has previously described the use of copper(I) complexes containing tris(pyrazolyl)borate ligands for the direct oxidation of C–H bonds of benzene as well as of alkanes,^7,8^ employing hydrogen peroxide as the oxidant (Scheme 1c). Some of those Tp^x^Cu(L) complexes are also capable of reacting with dioxygen and promoting the intramolecular oxidation of C(sp^3^)–H bonds.^9^ On the basis of this, we wondered about the use of such copper complexes in the reaction of benzene and dioxygen under mild conditions, aimed at forming phenol and understanding the reaction mechanism. Herein, we report the results of such study, where we have found that phenol is obtained in a process involving ascorbic acid as the sacrificial oxidant. DFT studies allowed us to propose the active species for such oxidation and the nuclearity of relevant intermediates.

## Results and Discussion

### Benzene Hydroxylation with Molecular Oxygen Induced by Copper

Our previous studies^7,8^ on C–H bond oxidation reactions with H_2_O_2_ were performed with two complexes bearing trispyrazolylborate ligands, Tp^Br3^Cu(NCMe) and Tp*^,Br^Cu(NCMe). However, the latter displays high reactivity toward O_2_, which leads to decomposition in a few minutes, whereas the former needs heating under O_2_ for several hours to decompose. Therefore, we chose Tp^Br3^Cu(NCMe) (**1**) as the promoter of the reaction between benzene and phenol in a homogeneous phase. The probe experiment was set with a solution of benzene (1 mmol) and complex **1** (0.05 mmol, 5 mol %) in acetonitrile (6 mL) and water (8 mL). Since the process requires the addition of two electrons and two protons for each molecule of benzene converted into phenol, 1 mmol of ascorbic acid was added, acting both as a reducing agent and as a proton source. Table 1 contains the relevant experiments for this study. The reaction was first performed for 1 h (entries 1–3) at room temperature under 1, 20, and 40 bar of O_2_, only in the latter case observing the formation of products derived from benzene in substoichiometric amounts relative to the copper complex. In this case, phenol was detected in a 60% yield (referred to **1**) as the sole oxidated arene. Prolonged reaction times (entries 4 and 5) led to mixtures of compounds, derived from overoxidation processes, in which hydroquinone, benzoquinone, and *o*-cathecol were detected. Actually, after a 6 h reaction time at room temperature, no phenol was detected (entry 5), with hydroquinone being the major oxidated arene compound in the final mixture.

**Table 1:**
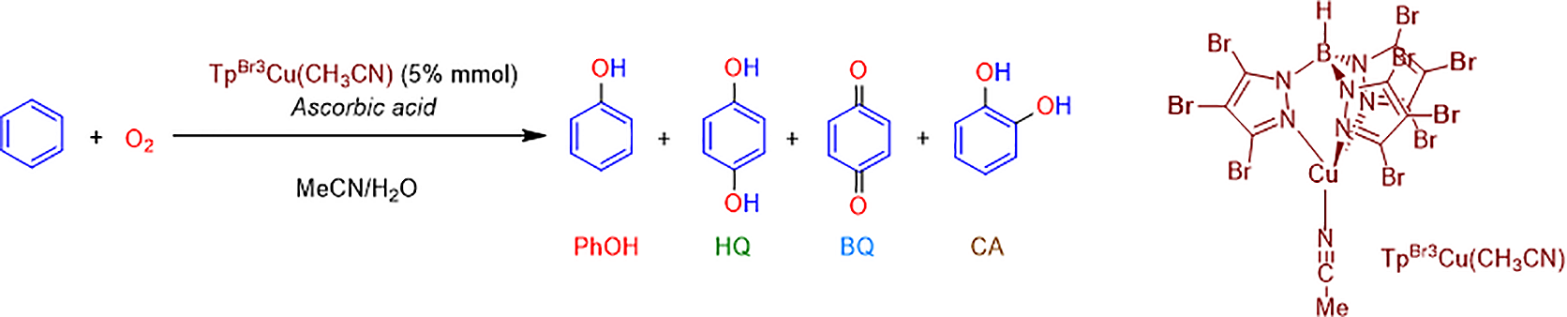
Copper-Mediated Hydroxylation of Benzene with O_2_ in the Presence of Ascorbic Acid^a^^,^^b^

entry	temp (°C)	pO_2_ (bar)	time (h)	phenol (PhOH)	hydroquinone (HQ)	benzoquinone (BQ)	*o*-cathecol (CA)
1	25	1	1				
2	25	20	1				
3	25	40	1	60%			
4	25	40	3	20%	20%	1.5%	<1%
5	25	40	6		40%	1.5%	10%
6	40	40	0.25	40%	8%	-	2%
7	40	40	0.75	40%	20%	4%	2%
8	40	40	1.5	20%	20%	8%	
9^c^	25	40	1				
10^d^	25	40	1				

a See Experimental Section for details.

b Yields are referred to the copper complex.

c No copper compound added.

d No ascorbic acid added.

The next variable changed was the temperature, which was set at 40 °C. Experiments run for 15, 45, and 90 min (entries 6–8) led to mixtures of the four compounds, i.e., phenol and overoxidation products. Blank experiments carried out in the absence of **1** or ascorbic acid were unproductive (entries 9 and 10). Therefore, the selectivity observed in entry 3 is lost when employing longer reaction times or higher temperatures. Regarding the ascorbic acid degradation, the major product observed by NMR was dehydroascorbic acid, as expected upon delivering electrons and protons. Despite the low yield, it is worth mentioning the simplicity of this system, employing a soluble copper complex^10^ and operating at room temperature, for the activation of O_2_ and oxidation of the C_sp2_–H bond of benzene.

All attempts to enhance the yield failed upon modifying the reaction conditions. We believe that this may be related to the decomposition of ascorbic acid under the reaction conditions since blank experiments revealed its degradation under the O_2_ atmosphere employed. However, ascorbic acid seems crucial for this transformation, and we have not found a surrogate for it. Thus, we have run experiments employing trifluoroacetic, citric acid, acetic acid, or sodium biphosphate under the same conditions in which ascorbic acid participated in the formation of phenol: none of them induced the oxidation of benzene to phenol at any extent. What makes ascorbic acid so special for this transformation? Why is this reaction stopped at the early stages? Given the importance of the benzene-to-phenol conversion, DFT studies have been performed to shed light on the mechanism of this transformation, aiming at designing more active systems in the future.

### DFT Studies: Generation of the Active Species

The nature of copper–oxygen active species for C–H/O–H activations in either biological or synthetic systems is controversial. Several mononuclear and binuclear copper–oxygen species arising from the interaction of copper(I) complexes with O_2_ have been proposed.^6,11^ Clear identification of such species in the reaction medium is usually very challenging, as it happens in our system. Computational studies can help to propose reactive intermediates, but careful exploration of all possible active species is mandatory. Calculations are aimed to clarify how putative oxidants are formed and to propose which is (are) responsible for attacking the substrate C–H bond. Suitable candidates should be radical species formed by means of reactions with low or moderate barriers, should have stabilities similar to reactants, and must be able to abstract a hydrogen atom from the substrate with a low or moderate reaction barrier.

**Scheme 2:**
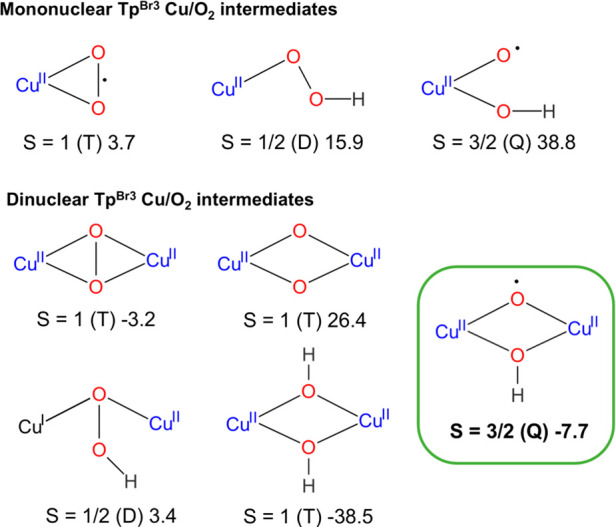
Mononuclear and Binuclear Copper–Oxygen Complexes Computationally Tested as Active Species for Benzene Hydroxylation with Their Spin State and Relative Gibbs Energy In kcal mol^–1^.

Scheme 2 summarizes the mono- and binuclear copper–oxygen species that have been computationally tested as potential benzene oxidants. Their relative Gibbs energies in acetonitrile with respect the reactants as well as their spin states are also displayed. For species containing hydrogen atoms, it is assumed that ascorbic acid (AAH_2_) is the hydrogen source. Accordingly, the energy of AAH_2_ and AAH^•^ has been added to estimate their relative energies. Optimized structures of all of them are collected in the Supporting Information (Figures S7 and S8).

### Mononuclear Copper Complexes

**Figure 1:**
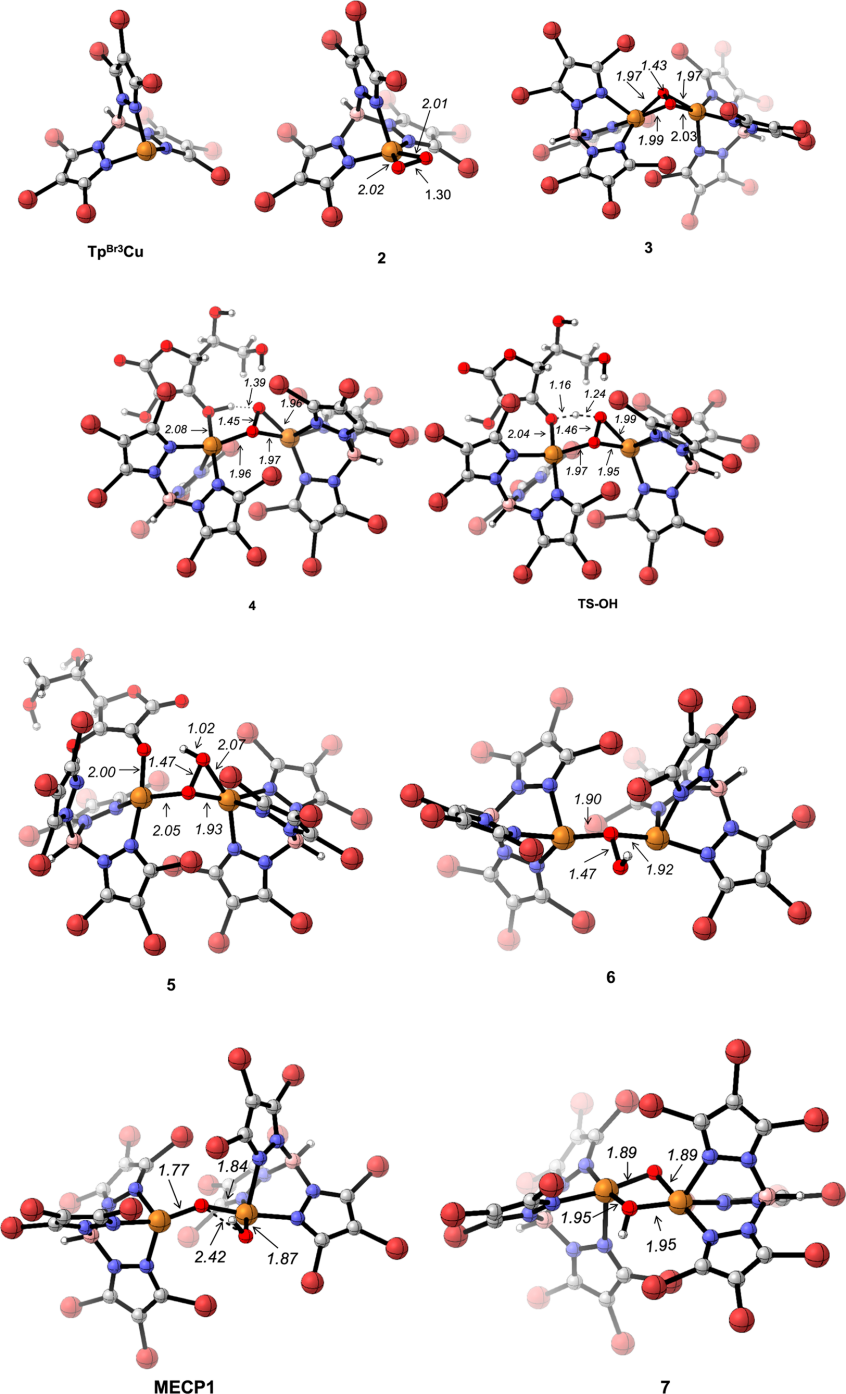
Optimized structures along the pathway for formation of the active species **7** (Gibbs energy profile in Figure 2).

Dioxygen activation by mononuclear copper(I) complexes may generate various copper–oxygen intermediates, such as Cu(II)–superoxo, Cu(II)–hydroperoxo, and Cu(II)–oxyl (Scheme 2).^11b^ The reaction starts from Tp^Br3^Cu(NCMe) (**1**), which can easily dissociate the acetonitrile ligand (Δ*G* = 11.3 kcal mol^–1^). This species can uptake O_2_ in a slightly endergonic process (Δ*G* = 3.7 kcal mol^–1^), in agreement with the high pressure of O_2_ required for its formation (Table 1). Tp^Br3^Cu(O_2_) (**2**) is a side-bound dioxygen adduct (Figure 1), structurally similar to the recently characterized (E^Mind^L)Cu(O_2_) (E^Mind^: 1,1,7,7-tetraethyl-1,2,3,5,6,7-hexahydro-3,3,5,5-tetramethyl-sindacene), described as a Cu^II^(O_2_^•–^) cupric superoxide.^12^ However, the dioxygen ligand in **2** is less activated (O–O = 1.30 Å) than in that in (*E*^Mind^L)Cu(O_2_) (O–O = 1.35 Å). Species **2** has a triplet ground state, therefore also displaying a Cu^II^(O_2_^•–^) nature. The singlet state Cu^III^(O_2_^2–^) is found 15.1 kcal mol^–1^ above the triplet. A side-on Cu(II)–superoxo complex was also characterized with the Tp^*t*Bu,*i*Pr^ ligand.^13^

Both side-on^14^ and end-on^15^ mononuclear Cu^II^–O_2_ species have been invoked as active species for hydrogen-atom abstraction of substrate C–H bonds. Accordingly, we considered **2** as a possible active species for hydrogen-atom abstraction (HAA) along the triplet spin surface, but we must discard this possibility: when the H atom approaches the oxygen ligand, no transition state is found; the energy increases steadily. Separated products of the HAA reaction (Cu^II^–OOH and Ph^•^) are found 52.3 kcal mol^–1^ above the reactants, making this pathway unfeasible.

As mentioned in the Experimental Section, ascorbic acid is present in the reaction medium, either in the neutral form AAH_2_ or in the deprotonated form AAH^–^ (p*K*_a_1 of AAH_2_ = 4.17). In this scenario, starting from Cu–superoxo species, there may exist two competing pathways. One is HAA from ascorbic acid (AAH_2_) or ascorbate anion (AAH^–^), and the other one is addition of a second Cu(I) complex to form a bimetallic species (to be discussed in the next section). Abstraction by **2** of a hydrogen atom from a O–H bond of ascorbic acid (AAH_2_) is much more favorable than that from the C–H bond of benzene. Ascorbate coordinates to the copper center during the process, inducing the displacement of the O_2_ ligand from side on to end on. This intermediate (SM1, Figure S10, Supporting Information) lies 15.1 kcal mol^–1^ above the separated reagents (Tp^Br3^Cu, O_2_, and AAH_2_) and in an almost barrierless process affords the Tp^Br3^Cu^II^(OOH)(AAH) triplet species (at 17.0 kcal mol^–1^ SM3, Figure S10, Supporting Information). Formation of the copper(II)–hydroperoxo species is even more favorable with the ascorbate anion. The initial intermediate [Tp^Br3^Cu^II^(O_2_)(AAH)]^−^ is 3.3 kcal mol^–1^ below Tp^Br3^Cu, O_2_ (SM1′, Figure S10) and AAH^–•^ and transfers one hydrogen to O_2_ with a barrier of only 4.3 kcal mol^–1^ (SM1-TS′, Figure S10). After dissociation of the ascorbate radical anion, the Cu(II)–hydroperoxo complex is formed, 7.6 kcal mol^–1^ above the separated reagents. Energetically, formation of this copper(II)–hydroperoxo species could not be discarded. Therefore, we checked whether it could be the active species that forms the phenyl radical. However, products from the HAA process are 46.6 kcal mol^–1^ above the reactants. We also performed the HAA reaction with the doublet species Tp^Br3^Cu^II^(OOH) arising from dissociation of AAH^•^ (Scheme 2). This pathway must be ruled out as well: the transition state for hydrogen abstraction is found to be 54.5 kcal mol^–1^ above the reactants (SM4-TS, Figure S10, Supporting Information).

We also considered the possibility of the involvement of copper(II)–oxyl species. Such complex can be formed by breaking the O–O bond in the Cu^II^–(OOH) complex. The resulting Cu^II^(O^•^)(OH) intermediate has been computed (Scheme 2, see also Figure S7 in the Supporting Information). It has a quartet spin state and is 38.8 kcal mol^–1^ above the reactants, an energy that allows discarding it as an active species.

Further details about the mononuclear pathways checked are given in the Supporting Information. We conclude that no one of the mononuclear complexes tested (copper(II)–superoxo, copper(II)–hydroperoxo, and copper(II)–oxyl) is able to abstract a hydrogen atom from benzene. Therefore, all of them must be discarded as active species in the benzene hydroxylation by Tp^Br3^Cu cores.

### Binuclear Copper Complexes

Interaction of a second unit of the Tp^Br3^Cu(I) complex with the copper(II)–superoxo **2** yields a μ-η^2^:η^2^-peroxodicoopper(II) complex **3** (Scheme 2) in a thermodynamically favored process: **3** is 3.2 kcal mol^–1^ more stable than the separated reactants. This stabilization is very similar to that attained by coordination of AAH^–^ to the mononuclear copper(II)–superoxo complex. However, energetically the ulterior evolution of the bimetallic complex **3** is very different from that of the mononuclear copper(II)–hydroperoxo. The optimized geometry of **3** (O–O = 1.43 Å; Cu–O_avg_ = 1.99 Å, Figure 1) is similar to that experimentally determined for [(Tp^*i*Pr,*i*Pr^)_2_Cu^II^_2_(O_2_)] (O–O = 1.41 Å; Cu–O_avg_ = 1.91 Å).^16^ Species **3** has a triplet ground state and thus a diradical character, potentially suitable to abstract a hydrogen atom from a C–H bond. Its singlet isomer **3**^**S**^ is 5.6 kcal mol^–1^ above the triplet. However, **3** cannot be the active species: HAA from benzene by **3** gives an intermediate 36.1 kcal mol^–1^ above the reagents (SB1, Figure S11, Supporting Information).

Interconversion of μ-η^2^:η^2^-peroxodicopper(II) complexes to the isomeric bis(μ-oxo)dicopper is very common and has been extensively studied.^17^ O–O breaking in **3**^**S**^ affords the Cu^III^(μ-O)_2_Cu^III^ complex (at 19.0 kcal mol^–1^) (SB2, and Figure S11, Supporting Information). Its triplet form Cu^II^(μ-O)_2_Cu^II^ is 26.4 kcal mol^–1^ above the reactants (Scheme 2 and Figure S8 in the Supporting Information). However, in the presence of ascorbic acid, this is not the favored evolution of **3**. Instead of transforming into the bis(μ-oxo)dicopper, a facile hydrogen-atom abstraction from a O–H bond of AAH_2_ generates a bimetallic hydroperoxo complex. As found for the same process in the mononuclear complex, AAH_2_ initially coordinates to one copper center (intermediate **4**, Figure 1) and then in a practically barrierless HAA the hydroperoxo complex **5** is formed (Figure 1).^18^ This reaction occurs in the triplet potential energy surface. Dissociation of AAH^•^ yields the doublet species **6**, which we describe as Cu^I^(μ-OOH)Cu^II^ (see spin analysis in the Discussion). In **6**, the bridging hydroperoxo ligand is bound to both copper centers through its proximal oxygen (Figure 1). Similarly to the mononuclear species, we also considered the anionic form AAH^–^ as the species that takes part in the formation of the Cu^I^(μ-OOH)Cu^II^ intermediate. The obtained energy profile has the same shape, although the relative Gibbs energies of all of the species are lowered (Figure S9, Supporting Information). Species **6** is placed only 3.4 kcal mol^–1^ above the reactants (taking AAH_2_) or 4.9 kcal mol^–1^ below (with AAH^–^). However, abstraction of a benzene C–H hydrogen by this species involves a rather high barrier of 28.8 kcal mol^–1^, discarding its role as an active species (SB3-TS, Figure S11, Supporting Information).

**Figure 2:**
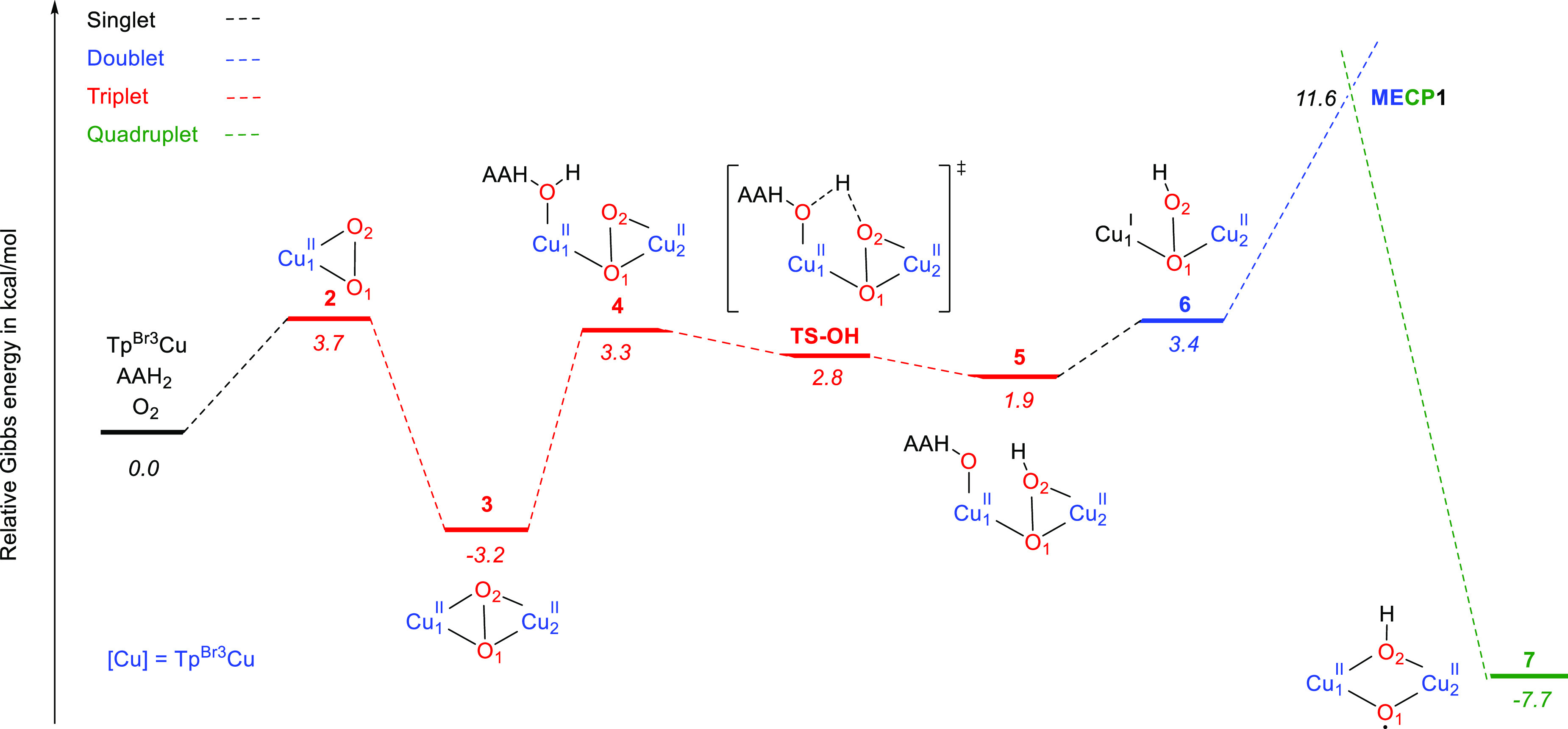
Gibbs energy profile for formation of active species **7** with neutral ascorbic acid as a hydrogen-atom donor. Cu stands for Tp^Br3^Cu. Relative Gibbs energies in acetonitrile in kcal mol^–1^.

Cleavage of the O–OH bond of the μ-hydroperoxo ligand **6** turns into a 5.4 kcal mol^–1^ more stable species **7**, containing one μ-O^•^ ligand and one μ-OH ligand. The transition state for O–O bond breaking in the doublet PES is 10.2 kcal mol^–1^ above **6** (SB4-TS, Figure S11, Supporting Information). The computed doublet structure of **7** (SB5, Figure S11, Supporting Information) has a broken symmetry nature and, in fact, involves three unpaired electrons (two alpha and one beta). Indeed, **7** is 5.7 kcal mol^–1^ more stable as a quartet (Figure 1) than as a doublet, and **6** to **7** interconversion takes place more easily by changing the spin state from doublet to quartet. The minimum energy crossing point between both potential energy surfaces (**MECP1**, Figure 1) has been located, 8.2 kcal mol^–1^ above **6**. Species **7** lies 7.7 kcal mol^–1^ below the reactants, has a quartet spin state, and can be described as a Cu^II^(μ-O^•^)(μ-OH)Cu^II^ complex. The bridging oxygen has a strong oxyl character (Mulliken spin population of 1.18). It is formed by a sequence of steps involving low barriers (see the Gibbs energy profile for its formation in Figure 2). As we will show in the next section, this species is able to perform H abstraction from benzene with a low barrier. Hence, **7** fulfills the required conditions to be the active species able to oxidize benzene formed in the reaction medium.

Computing accurate energy differences between different spin states by DFT methods is challenging: results can be highly dependent on the functional, particularly on the percentage of Hartree–Fock exchange in the functional.^19^ All of the energies reported herein have been obtained with the hybrid meta-GGA TPSSh functional supplemented with Grimme’s dispersion correction D3 using an extended basis set (basis-II, BS2, see the Computational Details). This methodology has proven to provide very good results predicting both the correct ground state and the right energy window for spin-crossover on first-row transition-metal complexes.^20^ Nevertheless, to assess the reliability of the doublet–quartet crossing, the relative energies of the doublet (**6**) and quartet (**7**) intermediates (referred to reactants) have been computed with 12 functionals containing variable percentages of Hartree–Fock exchange using an extended basis set (BS2). Although this energy difference is very dependent on the functional, all of the functionals but one agreed in showing **7** to be more stable than the reactants and more stable than **6** as well, assessing the proposal of **7** as the active species (see Table S1 in the Supporting Information). We also tested the possibility of abstraction of a O–H hydrogen atom of AAH_2_ by **7**, which easily leads to the Cu^II^(μ-OH)_2_Cu^II^ triplet complex. Indeed, this is a very stable species (Scheme 2, Figure S8 in Supporting Information), but we found that this complex is not able to abstract a hydrogen atom from benzene.

Summarizing this section, extensive exploration of potential mononuclear and dinuclear copper–oxygen complexes as active species for benzene oxidation led us to propose a Cu^II^(μ-O^•^)(μ-OH)Cu^II^ complex **7** as such species. In the next section, we will analyze benzene oxidation to phenol promoted by this complex.

### DFT Studies: Mechanism of Benzene Oxidation

**Scheme 3:**
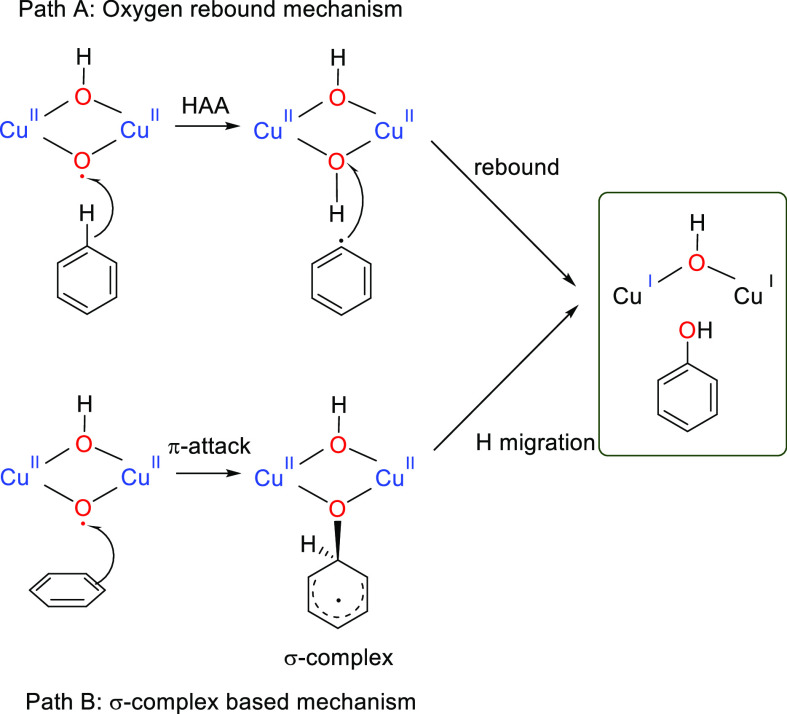
Possible Mechanisms for Benzene Hydroxylation Promoted by Copper–Oxyl Active Species **7**

The oxygen rebound mechanism is the most commonly proposed mechanism for copper–oxygen-mediated C–H activation processes.^11b,21^ In the rebound mechanism for C–H hydroxylation, an initial hydrogen-atom abstraction from the R–H substrate by a highly electrophilic species generates a substrate radical R^•^ and a metal hydroxide intermediate. In the subsequent rebound step, the organic radical attacks the M–OH center to give an alcohol group (Scheme 3, path A).^22^

**Figure 3:**
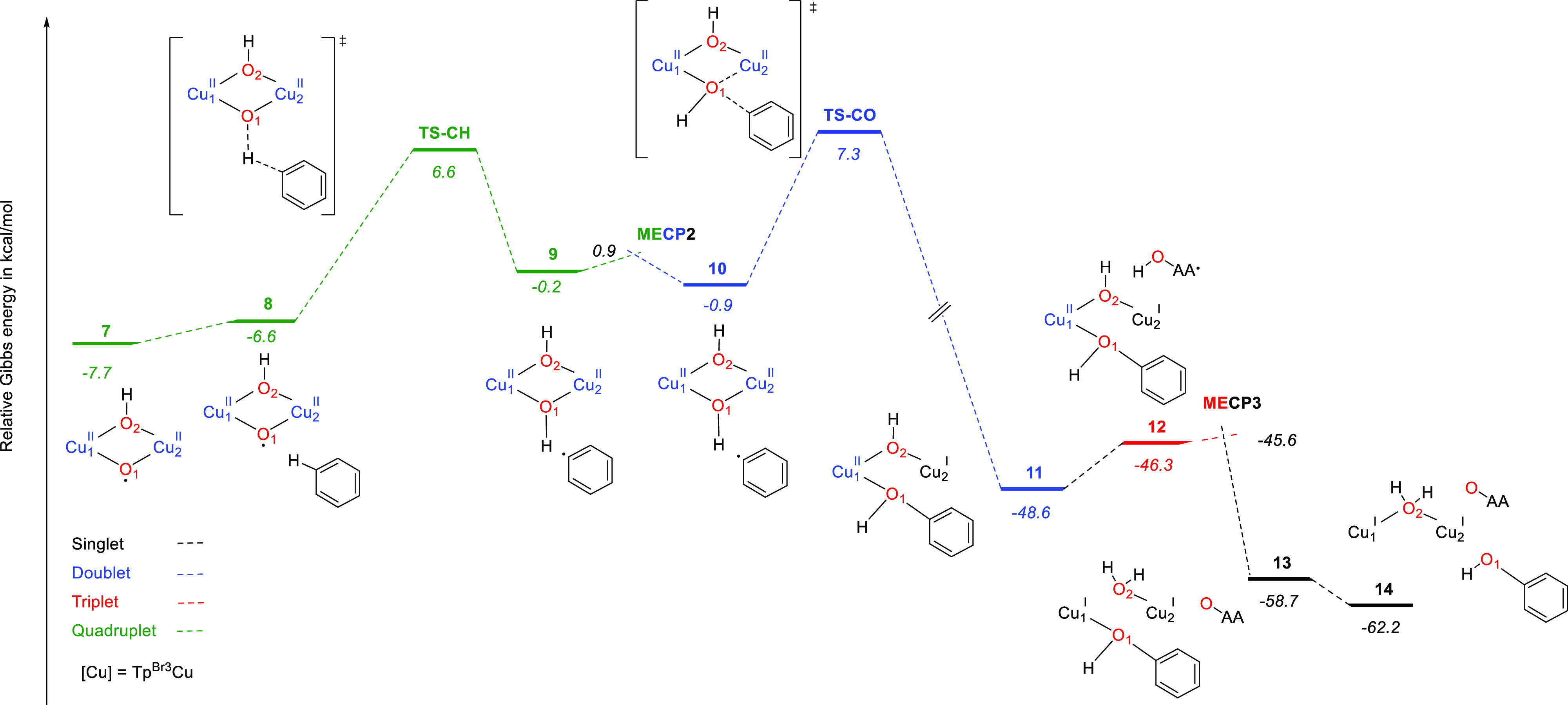
Gibbs energy profile for benzene hydroxylation promoted by **7** via an oxygen-rebound mechanism (path A, Scheme 3). Cu stands for Tp^Br3^Cu. Relative Gibbs energies in acetonitrile in kcal mol^–1^.

**Figure 4:**
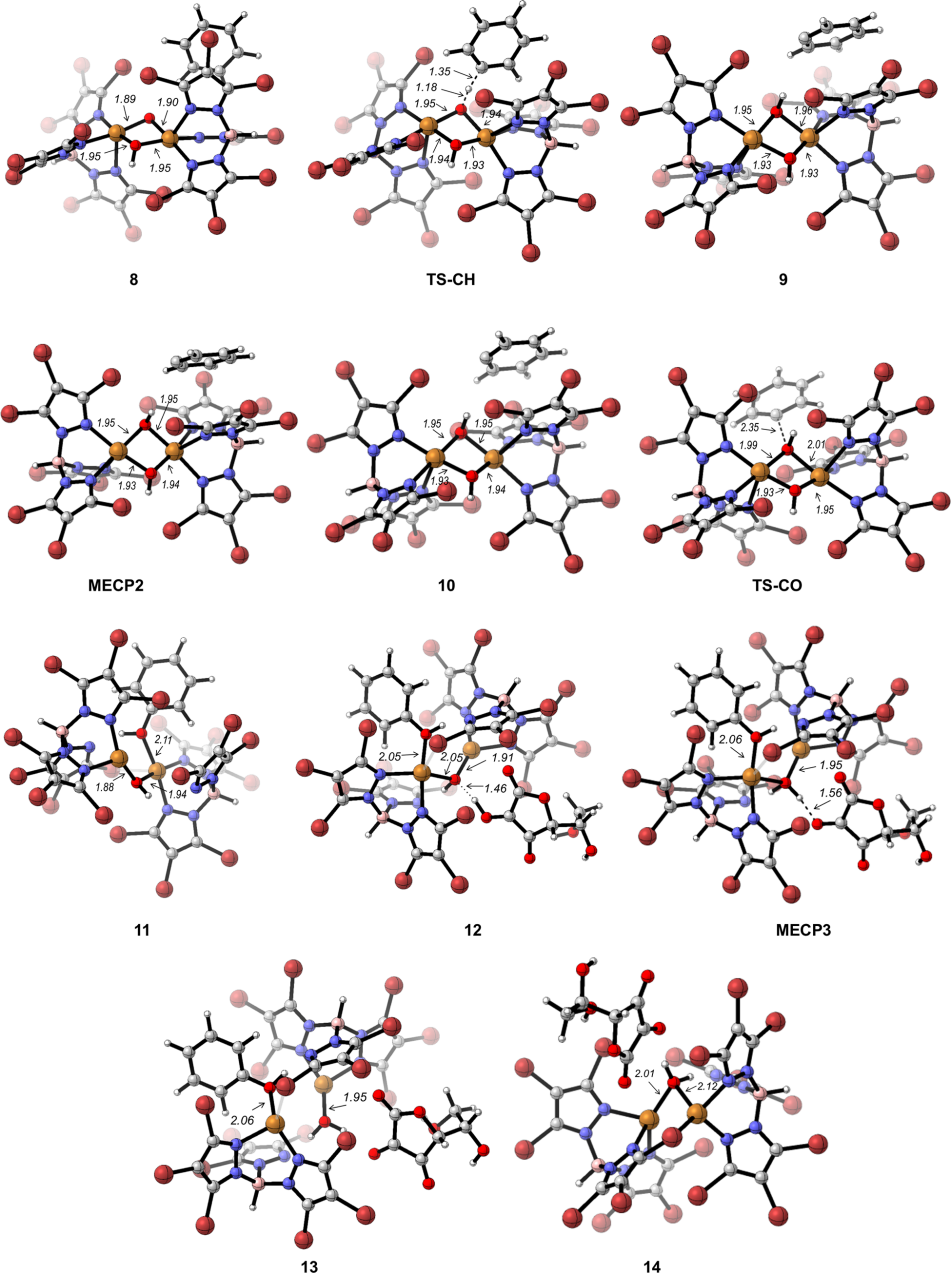
Optimized structures along the rebound pathway for benzene hydroxylation promoted by **7**.

The oxygen-rebound mechanism for benzene hydroxylation by the Cu–oxyl species **7** is initiated by the hydrogen-atom abstraction (HAA) from the substrate by the bridging oxyl to form the Cu_2_(μ-hydroxo)_2_ species and the carbon radical, and it is followed by a radical rebound step to build the C–O bond between the carbon radical and the Cu-bonded hydroxide. Figure 3 displays the Gibbs energy profile for benzene hydroxylation by **7** with an oxygen-rebound mechanism. The optimized structures of intermediates and transition states along the reaction pathway are shown in Figure 4.

Abstraction of a H atom from a benzene C–H bond takes place in the quartet PES through transition state **TS-CH** with a barrier of only 14.3 kcal mol^–1^ with respect to separated reactants (**7** + benzene) and affords intermediate **9** involving a Cu^II^(μ-OH)_2_Cu^II^ core and a phenyl radical. In **9**, three unpaired alpha electrons (quartet state) are found. As pairing of one alpha with one beta electron should occur in the formation of the C–OH bond, crossing to the doublet PES to form the broken symmetry doublet species **10** with two alpha and one beta electrons happens as well. The minimum energy crossing point between the quartet and the doublet PES (**MECP2**) was located 1.1 kcal mol^–1^ above **9**.

From **10**, coupling of the phenyl and O(H) radicals in the doublet PES forms the C–OH bond, after crossing transition state **TS-CO**. The Gibbs energy barrier for this step is low (8.2 kcal mol^–1^ from **10**). This step is highly exergonic and leads to complex **11**, with the phenol bonded to one copper center (Cu_1_) and a bridging hydroxo ligand (Figure 4).

In **11**, the oxygen of the μ-OH ligand keeps a radical character (Mulliken spin population 0.15) and can interact with AHH^•^ radicals that are present in the reaction medium (intermediate **12**, triplet PES, Figures 3 and 4). Crossing from the triplet to the singlet PES (through **MECP3**) entails transfer of a hydrogen atom from AAH^•^ to OH, forming a H_2_O ligand coordinated to copper (Cu_2_) (**13**, Figure 4) and dehydroascorbic acid. Release of the phenol product for Cu_1_ leads to a bimetallic copper(I)–copper(I) complex with a bridging water ligand (**14**).

**Figure 5:**
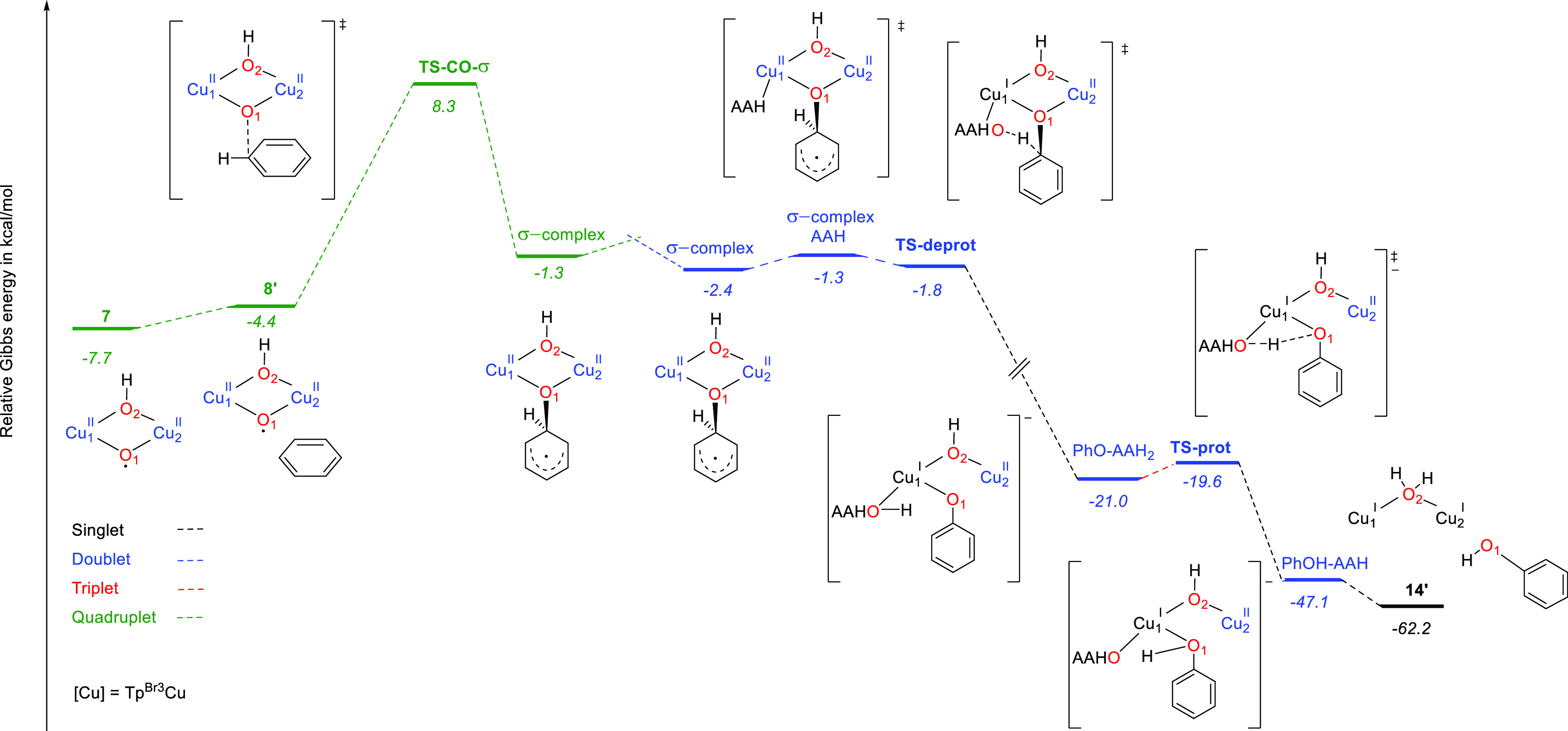
Gibbs energy profile for benzene hydroxylation promoted by **7** via a σ-complex mechanism (path B, Scheme 3). Cu stands for Tp^Br3^Cu. Relative Gibbs energies in acetonitrile in kcal mol^–1^.

**Figure 6:**
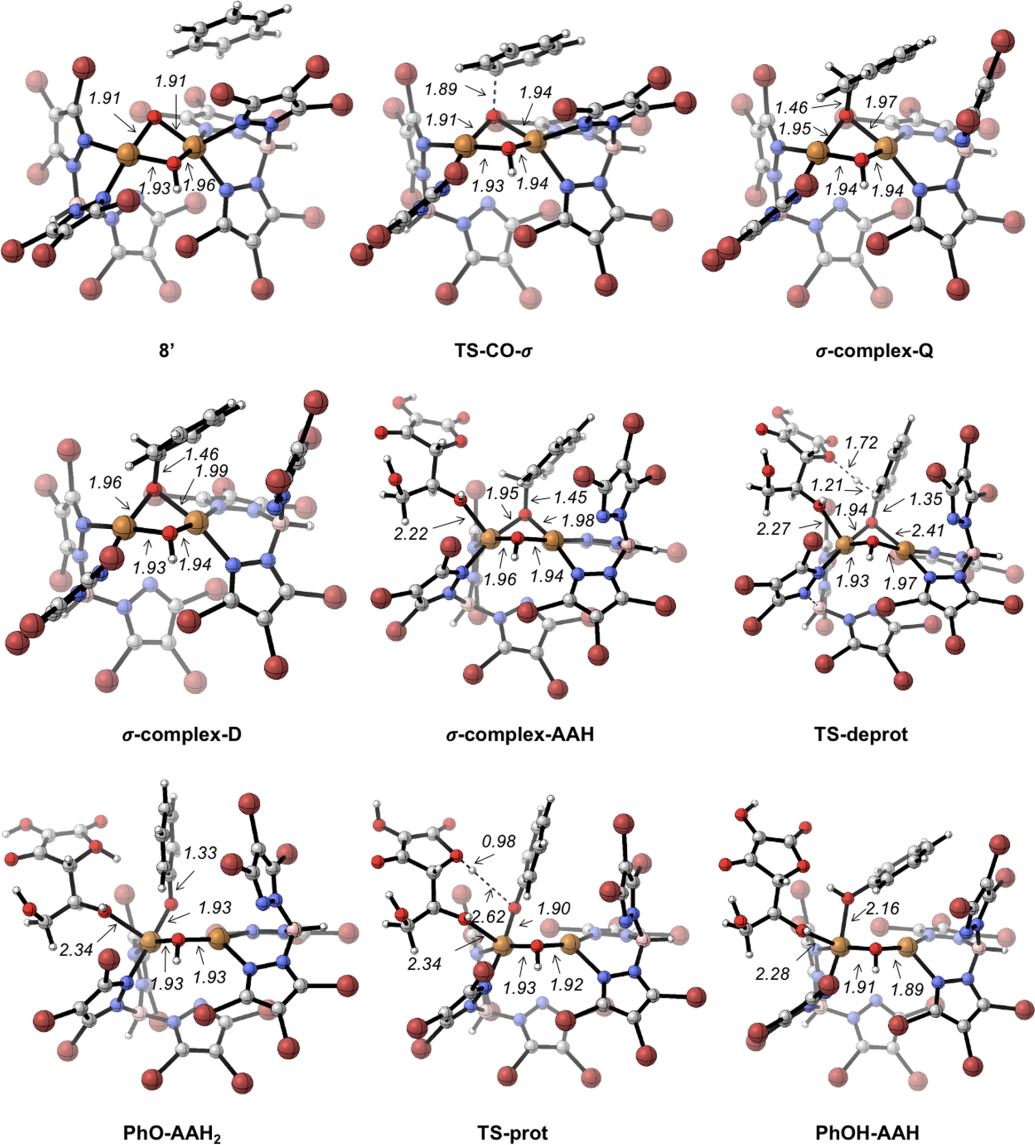
Optimized structures along the σ-complex pathway for benzene hydroxylation promoted by **7**.

An alternative mechanism has been proposed for arene hydroxylation catalyzed by cytochrome P450 enzymes. This mechanism involves an initial attack on the π system of the benzene by the electrophilic species of oxyl nature to produce a σ complex. In a second step, a proton shuttle, mediated in P450 by the porphyrin ring, transfers the proton from the ipso carbon to the oxygen, yielding the phenol (Scheme 3, path B).^23^ We also assessed the viability of the σ-complex-based mechanism for the benzene hydroxylation promoted by active species **7**. The Gibbs energy profile for benzene hydroxylation by **7** with a σ-complex-based mechanism is shown in Figure 5. Optimized structures of intermediates and transition states along this path are depicted in Figure 6. The electrophilic attack of the copper–oxyl on the π system of benzene takes place with a rather low barrier (16.0 kcal mol^–1^, **TS-CO-σ**, Figures 5 and 6). In this step, which occurs in the quartet potential energy surface, one electron has been pulled off from the π system by the oxyl, giving a radical character to the benzene ring. The resulting σ complex is slightly more stable as a broken symmetry doublet species. The proton in the ipso carbon of the σ complex must end up in the C–O oxygen to form the phenol. We found that an ascorbate anion can play the same role of proton shuttle for this hydrogen migration as one of the nitrogen atoms of the porphyrin ring plays in P450. Proton migration happens in the doublet potential energy surface. The ascorbate anion coordinates one copper center and, in an essentially barrierless process (**TS-deprot**, Figures 5 and 6), deprotonates the ipso carbon, yielding a phenoxy anion and reducing one copper center to Cu^I^ (**PhO-AAH2**, Figures 5 and 6). With a very low barrier (**TS-prot**, Figures 5 and 6), the proton is transferred from the O–H group of aspartic acid to the phenoxy oxygen, yielding the phenol product coordinated to a copper center (**PhOH-AAH**). From this intermediate, release of an AAH^•^ radical gives a [Cu^I^(μ-OH)Cu^I^]^−^ complex and the phenol.

The σ-complex-based mechanism entails an overall barrier of 16.0 kcal mol^–1^, only slightly higher than that of the oxygen-rebound mechanism (14.3 kcal mol^–1^), showing that both mechanisms could be competitive for benzene hydroxylation by active species **7**. In summary, computation of the reaction pathway for benzene oxidation to phenol provides evidence of the role of Cu^II^(μ-O^•^)(μ-OH)Cu^II^ complex **7** as the active species that promotes this reaction.

### Spin Density Changes along the Reaction Pathway

**Figure 7:**
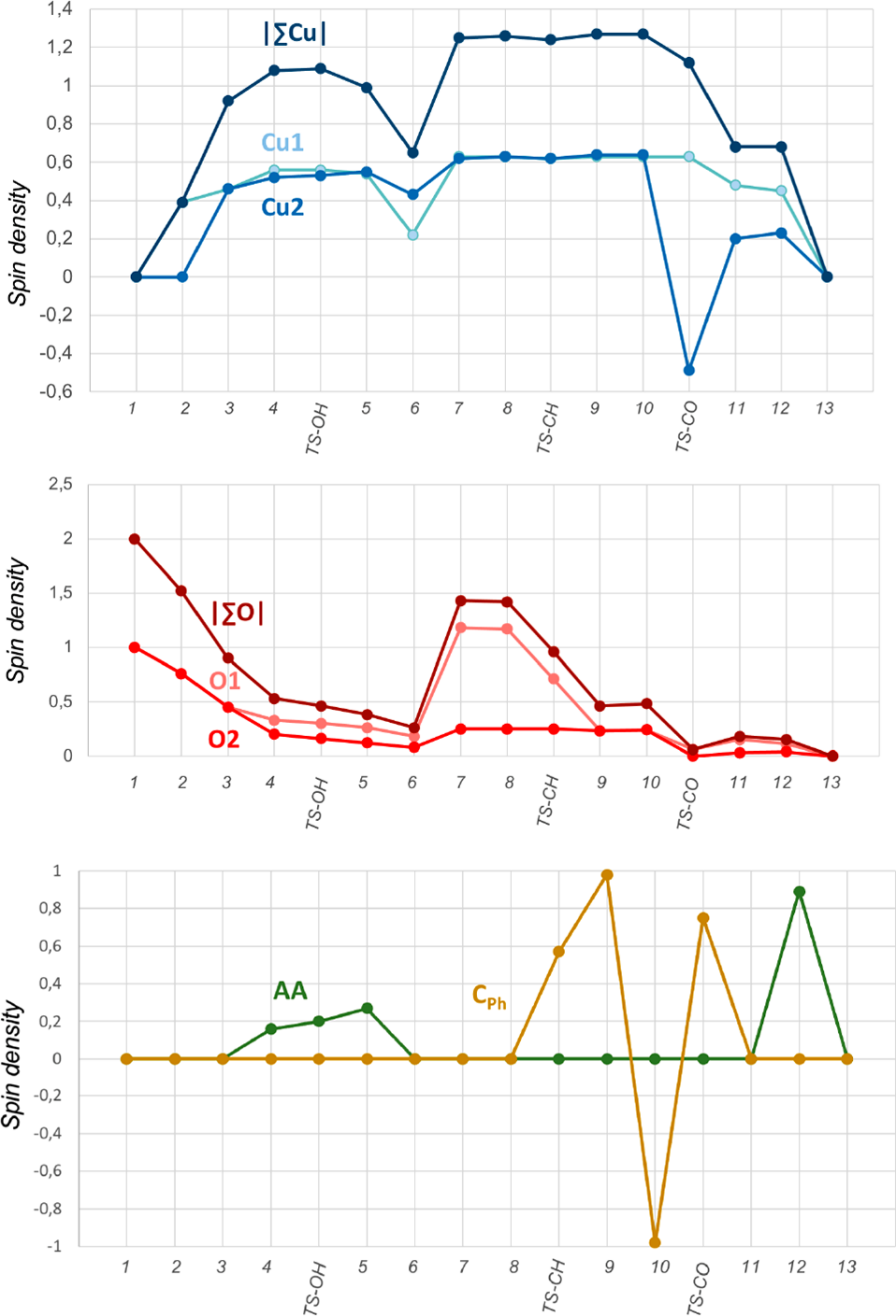
Evolution of Mulliken spin densities over the course of the reaction.

In the previous sections, we tentatively described the species involved in the reaction mainly from structural parameters and in terms of formal oxidation states. In this section, we will look more closely at the spin state changes along the reaction pathway to discuss more in depth the reaction mechanism. The oxidation state is a formal concept, very useful for a chemical description but hardly computable. We described Cu(II) as the copper centers with spin populations higher than 0.35e and Cu(I) as those with spin populations lower than 0.25e. Figure 7 displays the evolution of Mulliken spin populations along the generation of the active species (from **1** to **7**) and benzene hydroxylation (from **8** to **13**). All of the values can be found in the Supporting Information (Table S2).

O_2_ uptake by the copper(I) complex Tp^Br3^Cu causes spin polarization in copper, which is formally a monoxidation of copper to yield the Cu^II^–superoxo complex **2**, although spin density in the formal O_2_^–•^ unit is much larger (1.52) than in that copper (0.39). Coordination of the second copper(I) unit to **2** increases the spin density in the copper ions and decreases the spin density in the oxygen atoms. Species **3** is formally a Cu^II^(peroxo)Cu^II^ complex, although in fact there is one unpaired alpha electron shared between both copper centers and another unpaired electron shared between both oxygen atoms. Coordination of AAH_2_ to form **4** increases the radical character of both copper centers and decreases that of the oxygen atoms. Spin polarization in the coordinated AAH_2_ is very low (0.16). Ascorbic acid is transferring a proton to O_2_, thus decreasing its radical character (**TS–OH** and **5**). Spin density in both copper ions remains almost constant as well as in the ascorbic acid moiety. However, ascorbic acid dissociates as an AAH^•^ radical,^24^ inducing a reduction of Cu_1_ in **5**, which is a doublet complex, formally Cu^I^(μ-OOH)Cu^II^. Hydrogen-atom abstraction from the O–H bond of ascorbic acid involves stepwise proton transfer (PT)/electron transfer (ET) with mechanistically distinct PT and ET steps.^25^

**Figure 8:**
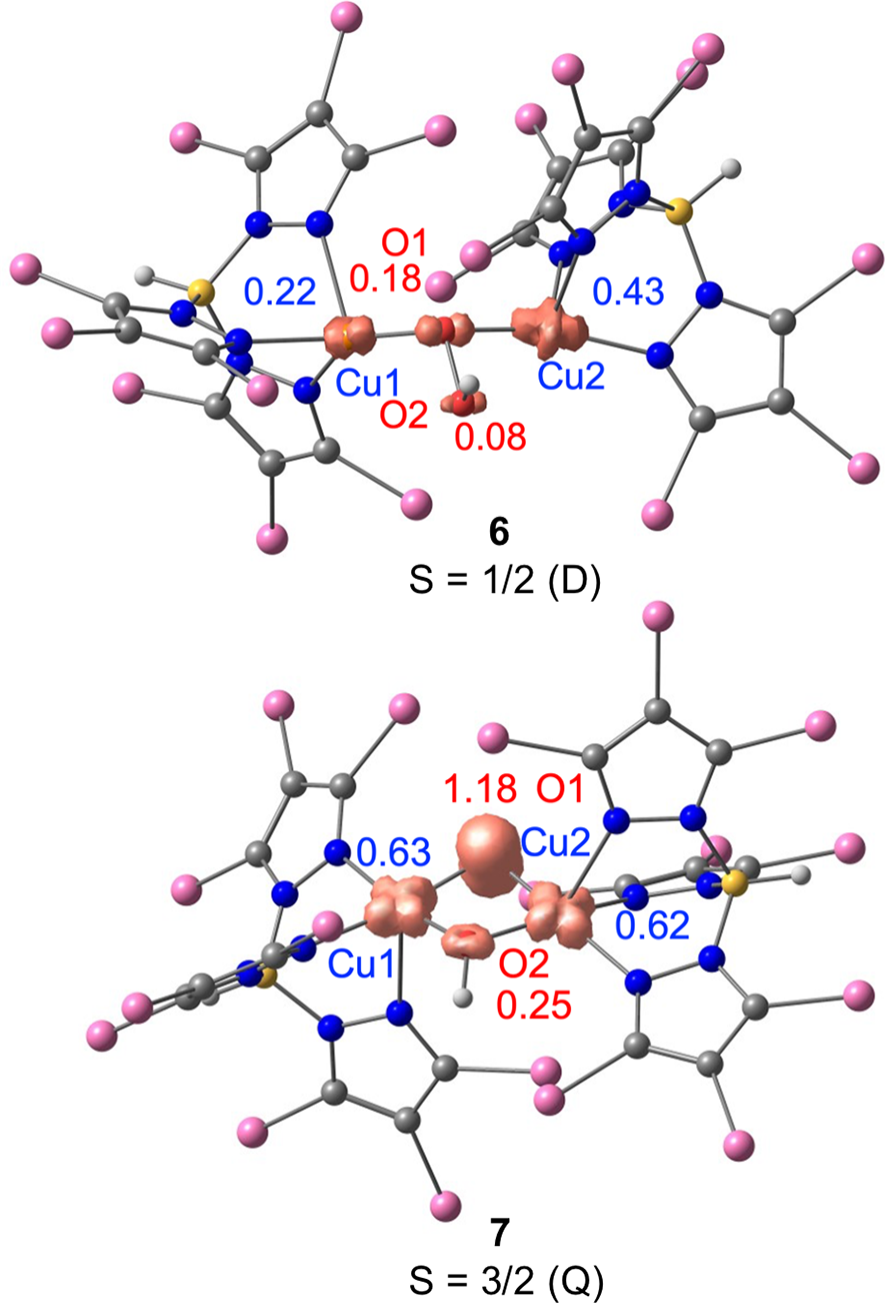
Spin-density plots and Mulliken spin populations of **6** and **7**.

The spin density of the bridging oxygen of the hydroperoxo ligand is low in **6** (0.18). However, this situation dramatically changes after breaking the O–OH bond. In **7**, with a quartet ground state, the bridging oxygen has a strong oxyl character (spin density = 1.18). This oxyl ligand will behave as a strong oxidant, able to abstract a hydrogen atom from strong C–H bonds. Spin density in the oxygen atom of the bridging hydroxo ligand is low (0.25), while that in the copper centers has increased. In this way, **7** is formally a Cu^II^(μ-O^•^)(μ-OH)Cu^II^ complex. Figure 8 shows spin-density plots for both species **6** and **7**, illustrating the changes that have occurred.

In the presence of benzene substrate, **7** abstracts a hydrogen atom from a C–H bond (**TS-CH** and **9**), generating a phenyl radical and significantly decreasing the spin density in the oxygen O1 that forms the O–H bond. The spin density in both copper centers remains constant at 0.63 (Cu_1_) and 0.64 (Cu_2_), corresponding to formally copper(II) ions. As commented above, the spin change of one electron, from alpha to beta, is required in order to couple unpaired electrons that will form the C–OH bond. This happens in the broken-symmetry doublet **10 (**spin density = −0.98 in C_Ph_ and 0.24 in O1), from which C–O bond formation starts, in a step usually called an oxygen-rebound mechanism. After formation of phenol, the spin density in both oxygens (0.15 in O1 and 0.03 in O2) and in Cu_2_ (0.20) has notably decreased in **11**. However, the remaining spin density in the oxygen atom of the O–H ligand of **11** (0.15) allows abstracting a hydrogen atom of a AAH^•^ radical in a triplet to singlet crossing that ends up with a water ligand bridging two copper(I) centers and a dehydroascorbic acid molecule (**13**).

We checked the hydrogen-abstracting capability of **7** with two other substrates with C_sp3_–H bonds: cyclohexane and methane. For the alkane substrates, only the oxygen-rebound mechanism (Path A, Scheme 3) can be at work. Indeed, **7** is able to abstract a hydrogen atom from both alkanes with transition states similar to that of benzene (see the TS geometries in the Supporting Information, Figure S12: **TS_CycHx** and **TS_Met**) and very low barriers: 4.4 and 12.8 kcal mol^–1^ with respect to **7** + alkane for cyclohexane and methane, respectively). Thus, **7** appears as the active species responsible for hydroxylation of substrates with strong C–H bonds. It is important to point out that this high-spin species is only stable in the presence of two copper centers. Whereas monometallic Cu^II^(O^•^)(OH) is found 38.8 kcal mol^–1^ above the reactants (Scheme 2), making it unreachable under the reaction conditions, bimetallic Cu^II^(μ-O^•^)(μ-OH)Cu^II^ is 7.7 kcal mol^–1^ below the reactants and can be formed through a sequence of steps having low barriers (Figure 2). However, the high oxyl character of **7**, responsible for its easiness to perform HAA from the C–H bond of substrates, can also be the reason for its deactivation. With ascorbic acid present in the reaction medium, **7** can perform the HAA from a O–H bond of AAH_2_ yielding the Cu^II^(μ-OH)_2_Cu^II^ triplet complex, which is very stable (Scheme 2, Figure S8 in Supporting Information), but is not able to abstract a hydrogen atom from benzene.

**Scheme 4:**
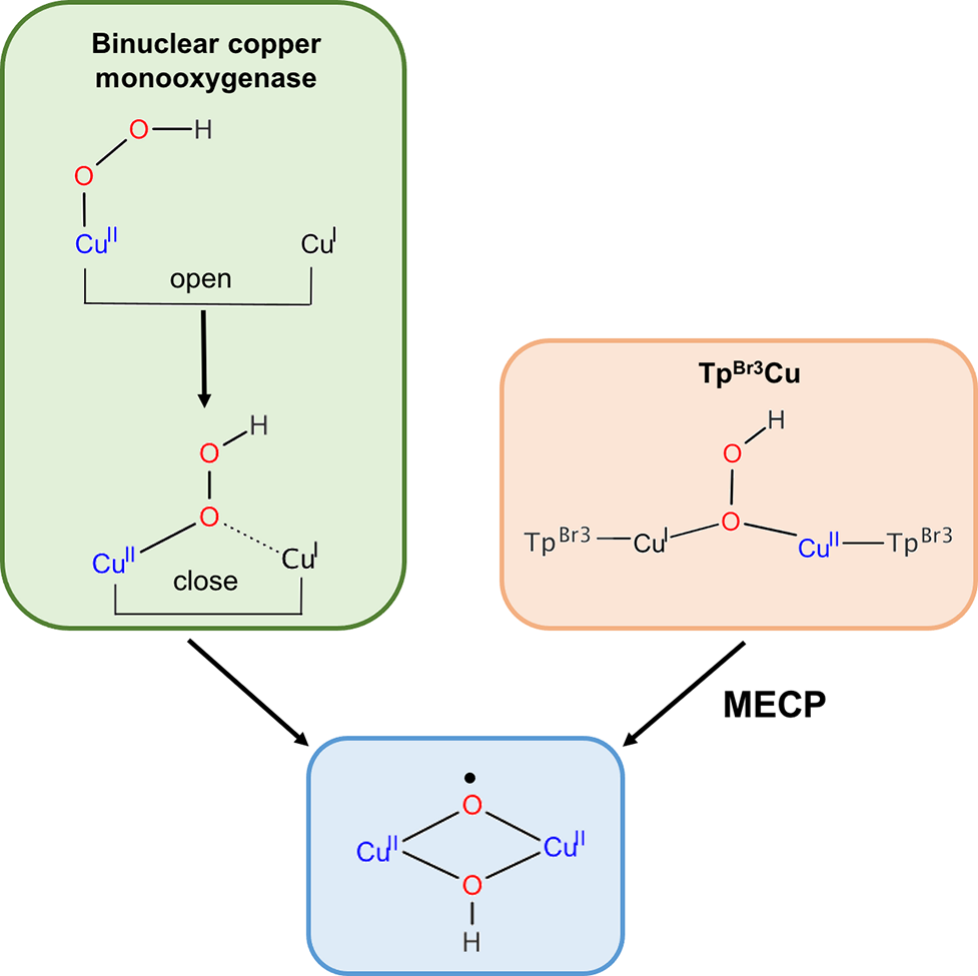
Formation of Active Species for C–H Bond Hydroxylation in Binuclear Copper Monooxygenases and Tp^Br3^Cu Complex

Very recently, a thorough computational study has afforded a new perspective for O_2_ activation and substrate hydroxylation by binuclear copper monooxygenases.^26^ As in our system, the proposed active species for substrate hydroxylation has a Cu^II^(μ-O^•^)(μ-OH)Cu^II^ nature. Scheme 4 compares the key steps for the formation of such species for binuclear copper monooxygenases with our results for Tp^Br3^Cu.

Although these enzymes contain two copper(I) cofactors very far away (about 11 Å, “open” conformation), this study showed that in the presence of cosubstrate ascorbate hydrogen-atom abstraction leads to the formation of a Cu(II)–OOH intermediate that is inert toward H abstraction from the substrate. However, by approaching the copper atoms (“closed” conformation), a stable binuclear Cu^I^(μ-OOH)Cu^II^ species affords the more stable species Cu^II^(μ-O^•^)(μ-OH)Cu^II^, which is the reactive intermediate responsible for substrate hydroxylation.^11b,26^ This is the same active species **7** we found for the hydroxylation of benzene promoted by Tp^Br3^Cu, proving for first time that it can also operate in synthetic systems. As for the enzyme, neither Cu^II^–superoxo nor Cu^II^(hydroperoxo) is reactive toward substrate hydroxylation.^22a^ Formation of the active species also follows a similar pathway. In our case, the bimetallic complex is formed by coordination of a Tp^Br3^Cu molecule to the initial Cu^II^(O_2_^–•^) intermediate, and from this point the bimetallic complex is kept. We also showed that the interconversion between Cu^I^(μ-OOH)Cu^II^ and Cu^II^(μ-O^•^)(μ-OH)Cu^II^ (from **6** to **7** in our system) that involves O–OH cleavage can take place easily by means of a spin-state crossing from doublet to quartet. Overall, the mechanism of substrate hydroxylation by the active species is the same as that in binuclear copper monooxygenases and in our Tp^Br3^Cu+O_2_ + AAH_2_ system.

## Conclusions

We experimentally proved that Tp^Br3^Cu(NCMe), in the presence of ascorbic acid, can promote the direct hydroxylation of a benzene C–H bond with O_2_ as the oxidant, yielding phenol as a main product. However, the reaction is stopped at the early stages, and benzene oxidation products are obtained in substoichiometric amounts relative to the copper complex. No reaction intermediate or the active species could be identified. Thus, we turned to DFT calculations to get information about this process that could help to improve it. A thorough exploration allowed us to propose a bimetallic Tp^Br3^Cu^II^(μ-O^•^)(μ-OH)Cu^II^Tp^Br3^ intermediate as the active species responsible for the oxidation process. Ascorbic acid is crucial for its formation as hydrogen-atom abstraction from the O–H bond of ascorbic acid (or ascorbate anion) in a stepwise proton transfer (PT)/electron transfer (ET) gives its precursor hydroperoxo complex Tp^Br3^Cu^II^(μ-OOH)Cu^I^Tp^Br3^, which is not active for HAA. Any mononuclear species is clearly unfeasible. Identification of the active species for C–H bond hydroxylations can pave the way for further preparation of more robust synthetic compounds that could catalyze such reactions.

## Experimental Section

### General Methods

Preparation of Tp^Br3^Cu(NCMe) was carried out using literature methods. Solvents were distilled and degassed before use. Oxidated arenes were purchased from Aldrich. HPLC studies were performed in an Agilent 1260 Infinity II. NMR spectra were recorded on an Agilent 400 MR or Agilent 500 DD2 device. PARR Micro Bench Top reactors were employed for the oxidation reactions.

### General Catalytic Experiment

The oxidation reactions were carried out in a 100 mL high-pressure reactor in which benzene (1 mmol), ascorbic acid (1 mmol), and the catalyst (0.05 mmol) were dissolved in an acetonitrile/water (3:1 v-v, 8 mL) mixture. The reactor was charged with O_2_ at 40 bar and was stirred for the desired time. After depressurization of the vessel, the reaction mixture was diluted to an exact volume with acetonitrile, and the solution was evaluated by HPLC using calibration curves. After that, for double checking, the volatiles were removed under reduced pressure and the crude was analyzed by ^1^H NMR spectroscopy using 1,3,5-trimethoxybenzene as an internal standard.

### Computational Methodology

Theoretical calculations were performed at the DFT level of theory using Gaussian09 software.^27^ The structures of all of the intermediates and transition states were optimized in acetonitrile solvent (ε = 35.688) with the SMD continuum model^28^ using the hybrid meta-GGA TPSSh functional^29^ supplemented with Grimme’s dispersion correction D3.^30^ Additional calibration calculations employing a set of functionals spanning a large range of percentages of Hartree–Fock exchange were carried out for certain structures (see Table S1 in the Supporting Information). Basis set BS1 was used for the optimizations. BS1 includes the 6-31G(d,p) basis set for the main-group elements^31^ and the scalar relativistic Stuttgart–Dresden SDD pseudopotential and its associated double-ζ basis set^32^ complemented with a set of f polarization functions for the copper atoms.^33^ Frequency calculations were carried out for all of the optimized geometries in order to characterize the stationary points as either minima or transition states. Connections between the transition states and the corresponding minima were checked by displacing in both directions, following the transition vector, the geometry of the transition states, and subsequent geometry optimization until a minimum was reached.

Gibbs energies in acetonitrile were calculated at 298.15 K, adding to the potential energies in acetonitrile, obtained with single-point calculations using an extended basis set (BS2) at the BS1-optimized geometries, the thermal and entropic corrections obtained with BS1. BS2 consists of the def2-TZVP basis set for the main-group elements and the quadruple-ζ def2-QZVP basis set for Cu.^34^ A correction of 1.9 kcal mol^–1^ was applied to all Gibbs values to change the standard state from the gas phase (1 atm) to solution (1 M) at 298.15 K.^35^ In this way, all of the energy values in the energy profiles are Gibbs energies in acetonitrile solution calculated using the formula
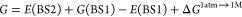
where Δ*G*^1 atm→1M^ = 1.9 kcal mol^–1^ is the Gibbs energy change for compression of 1 mol of an ideal gas from 1 atm to the 1 M solution-phase standard state.

To locate the minimum energy crossing points (MECP) between the singlet and the triplet potential energy surfaces, the program developed by the group of Harvey was employed.^36^ To confirm that the MECP connects the two intermediates located in the two energy surfaces, the MECP structure was optimized in the different spin states involved in the crossing. The Gibbs energies in solution of the MECP were estimated by adding to the calculated potential energy of the MECP thermal and entropic corrections calculated with the option freq = projected of the Gaussian 09 program.^37^ Three-dimensional structures were generated using CYLview.^38^

## Supporting Information Available

Experimental details and extended description of the computational results (PDF)
Cartesian coordinates of all the optimized structures (XYZ)

## Supplementary Material

om2c00202_si_001.pdf

om2c00202_si_002.xyz
