# Supplementary material for: Direct Benzene Hydroxylation with Dioxygen Induced by Copper Complexes: Uncovering the Active Species by DFT Calculations
Source: Organometallics. 2022 Jul 14;41(14):1892–904. doi: 10.1021/acs.organomet.2c00202 (PMC9344391; doi:10.1021/acs.organomet.2c00202)
Supplement: Supplementary file 1 — om2c00202_si_001.pdf [file om2c00202_si_001.pdf]

# SUPPORTING INFORMATION for

# Direct Benzene Hydroxylation with Dioxygen Induced by Copper Complexes: Uncovering the Active Species by DFT Calculations

*Elena Borrego,[a] Laura Tiessler-Sala,[b] Jesus J. Lázaro[c,]* Ana Caballero,[a,]* Pedro J. Pérez[a,]* Agustí Lledós[b,]**

[a]Laboratorio de Catálisis Homogénea, Unidad Asociada al CSIC, CIQSO-Centro de Investigación en Química Sostenible and Departamento de Química, Universidad de Huelva, Campus de El Carmen 21007 Huelva, Spain.

[b]Departament de Química, Universitat Autònoma de Barcelona, 08193 Cerdanyola del Vallès, Barcelona (Spain).

[c]Cepsa Research Center, Compañía Española de Petróleos S.A., Alcalá de Henares, Madrid, 28850, Spain

**Table of Contents**


| | | |
|---|---|---|
| 1 | Materials and methods | S2 |
| 2 | Representative NMR spectra | S2 |
| 3 | Representative HPLC chromatograms | S4 |
| 4 | 3D-views of mono- and binuclear copper oxygen species computationally tested as potential benzene oxidants (complexes in Scheme 2) | S6 |
| 5 | Gibbs energy profile for formation of active species with anionic ascorbate ($AAH^-$) in the hydrogen transfer step | S7 |
| 6 | Dioxygen activation and HAA from benzene by mononuclear copper(I) complexes | S8 |
| 7 | Dioxygen activation and HAA from benzene by binuclear copper(I) complexes | S9 |
| 8 | HAA from $C_{sp3}$-H bonds by active species **7** | S10 |
| 9 | DFT benchmark study of the relative stabilities of $Cu^{I}$-(μ-OOH)$_2$-$Cu^{II}$ (**6**, doublet) and $Cu^{II}$(μ-O·)(μ-OH)$Cu^{II}$ Cu (**7**, quartet) species | S11 |
| 10 | Spin densities | S12 |
| 11 | Absolute energies of computed species, in Hartrees | S14 |

## 1. Materials and Methods

**General Methods**. The chemicals were purchased from Aldrich and Alfa Aesar and were used without purification. The gaseous reactants, such as $O_2$ and Air, were obtained from Air Liquide and used as received. The water employed in the experiments was Mili-Q quality. Experiments under pressure were run in a PARR Micro Bench reactors with inside Teflon beaker. The beakers were treated with aqua regia after their use to avoid contamination with trace metals. The chemicals used in analytic studies presented HPLC and analytic grade. Calibration curves were built using commercial products. HPLC analyses were performed Agilent Technologies 1260 Infinity II equipped with a G7112B binary pump, a G4767A Diode Array Detector WR, a G7116A 1260 thermostat column compartment, a G4767A automatic multisampler and an Avantor® ACE® EXCEL® 3μm C18-AR column (150 x 4,6 mm). The mobile phase was a mixture of solvents A (ammonium acetate aqueous solution, pH= 4.4) and B (acetonitrile). A gradient program was used to give adequate resolution between the products. The gradient started from 5-10% (v/v) of B for 20 min., 10-36% (v/v) of B from 20-33 min and an isocratic elution with a composition of 10% A and 90% B were employed for the last 10 min. The liquid phase flow rate was set at 1 mL/min. The injection volumes were 10 μL and column temperature was 25 °C. The detector signals were 254 nm and 276 nm.

## 2. Representative NMR Spectra

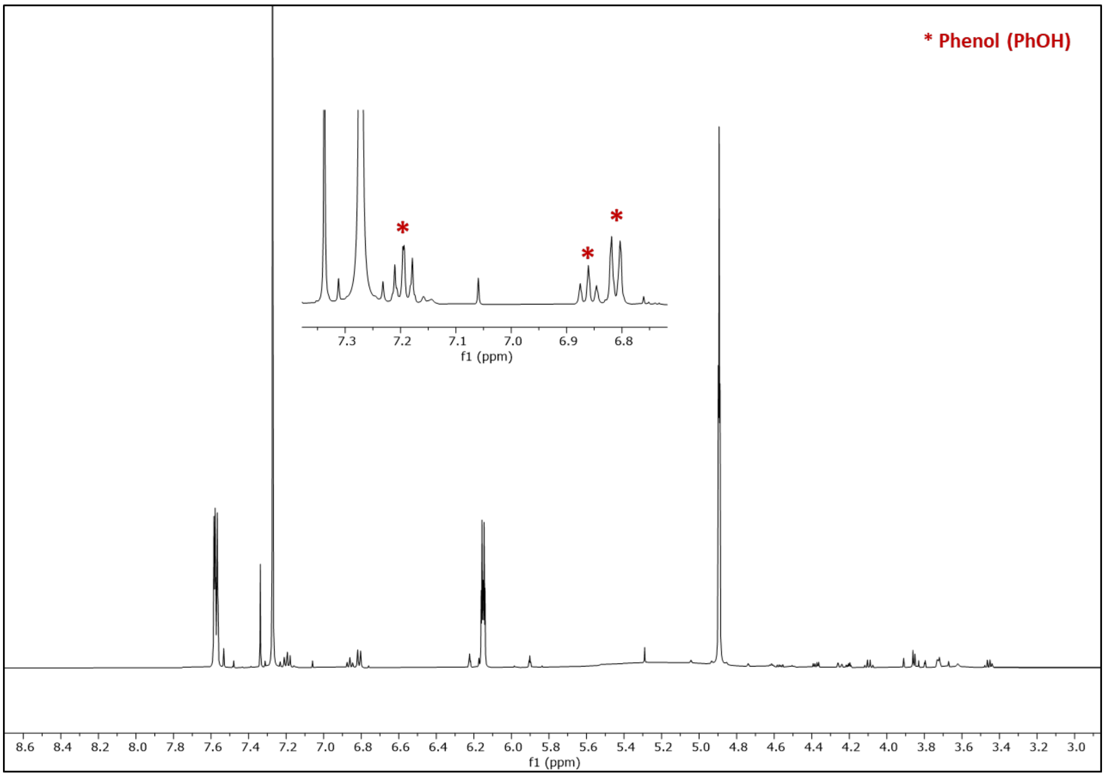

***Figure S1.*** *$^1$H NMR (400 Hz, $CDCl_3$) spectrum of crude reaction mixture for the benzene oxidation reaction in presence of $Tp^{Br3}Cu(NCMe)$ as catalyst under 40 bar $O_2$ at room temperature for 1 h.*

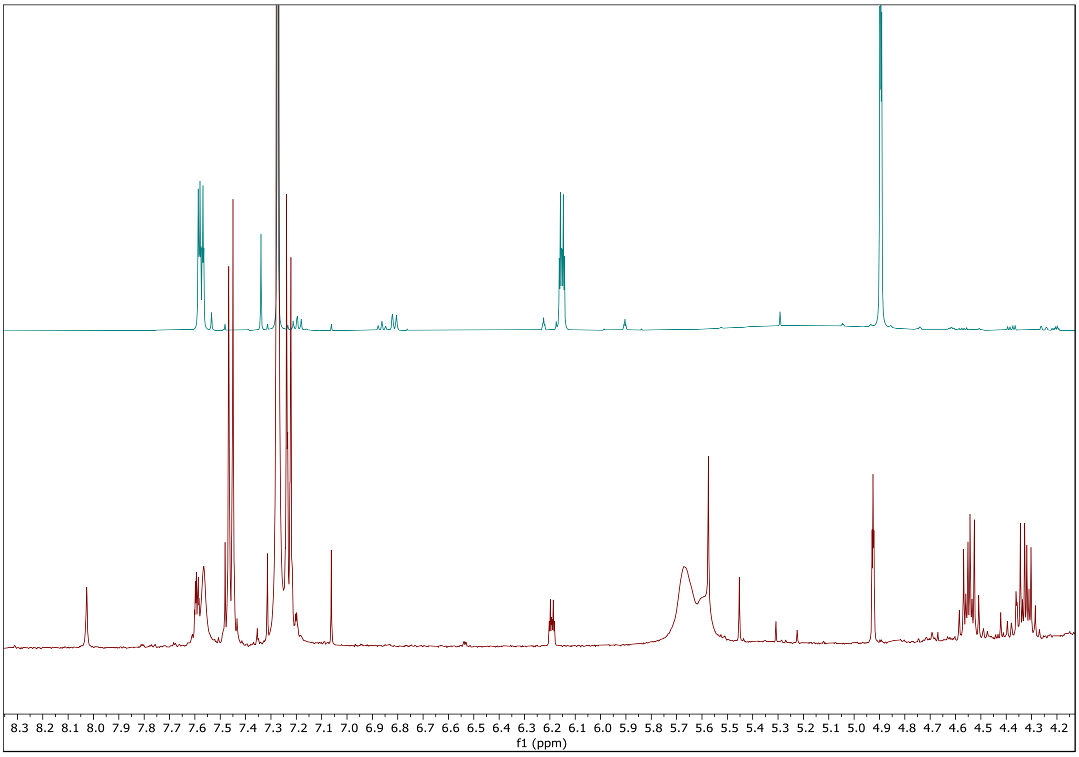


***Figure S2.*** *$^1$H NMR (400 Hz, $CDCl_3$) spectrum of crude reaction mixture for the benzene oxidation reaction in presence of $Tp^{Br3}Cu(NCMe)$ (top) and $^1$H NMR (400 Hz, $CDCl_3$) spectrum of crude reaction mixture in absence of benzene (bottom).*

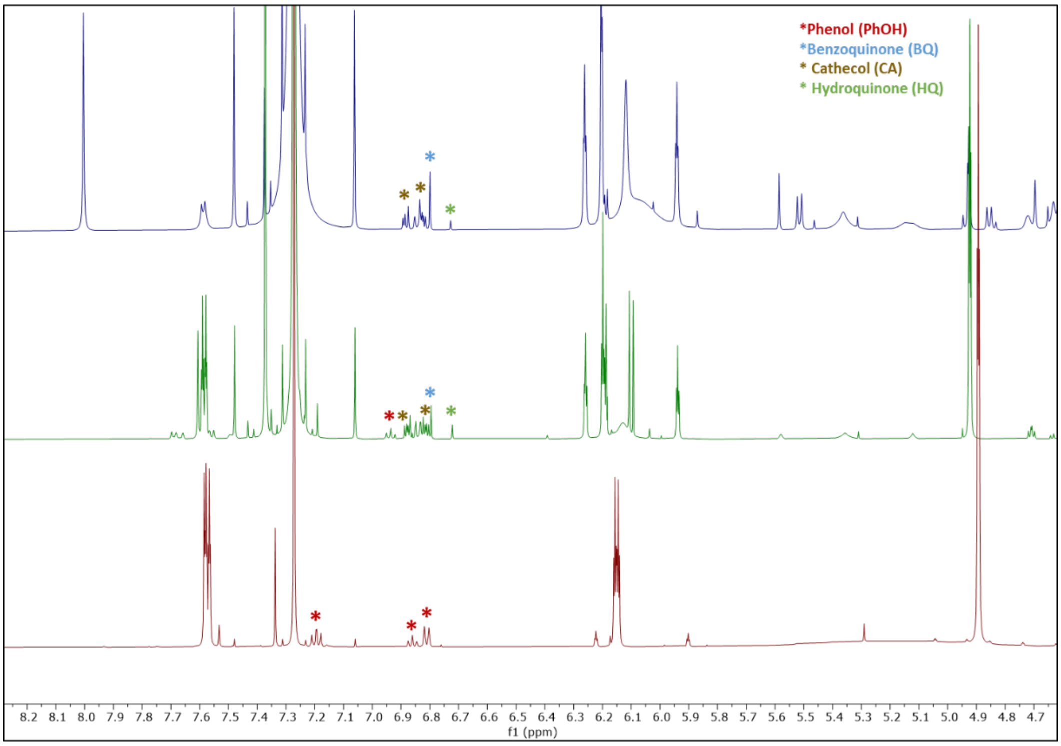

***Figure S3***. *$^{1}$H NMR (400 Hz, $CDCl_3$) spectrum of crude reaction mixture for the benzene oxidation reaction in presence of $Tp^{Br3}$Cu(NCMe) under 40 bar $O_2$ at room temperature for 6 h (top), 3 h (middle) and 1 h (bottom).*

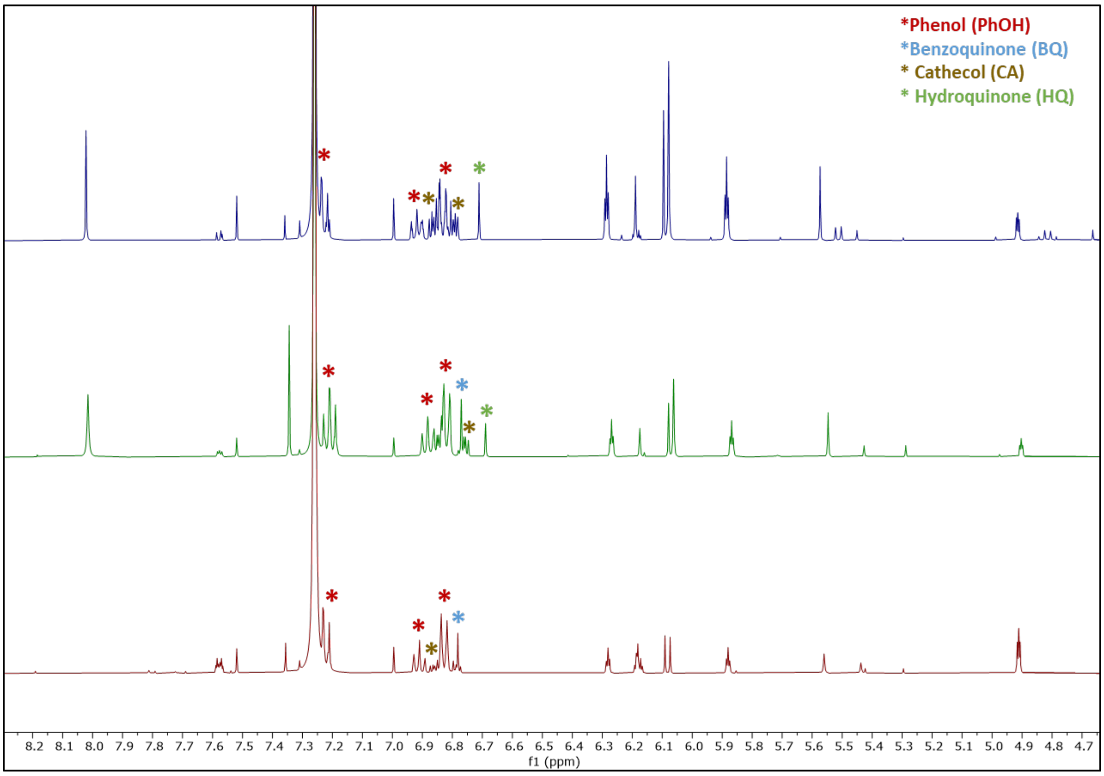


***Figure S4***. *$^{1}$H NMR (400 Hz, $CDCl_3$) spectrum of crude reaction mixture for the benzene oxidation reaction in presence of $Tp^{Br3}$Cu(NCMe) under 40 bar $O_2$ at 40 °C for 1.5 h (top), 0.75 h (middle) and 0.25 h (bottom).*

## 3. Representative HPLC chromatograms

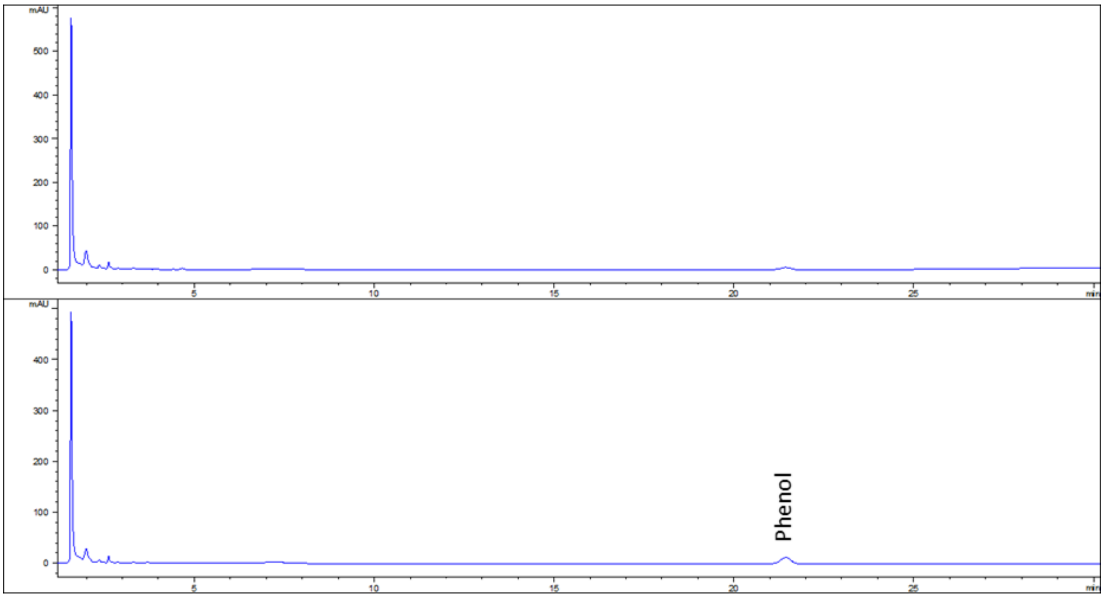

***Figure S5.*** *HPLC chromatogram of the benzene oxidation reaction in presence of $Tp^{Br3}Cu(NCMe)$ under 40 bar $O_2$ at room temperature for 1 h. Top: UV detector 254 nm. Bottom: UV detector 275 nm.*

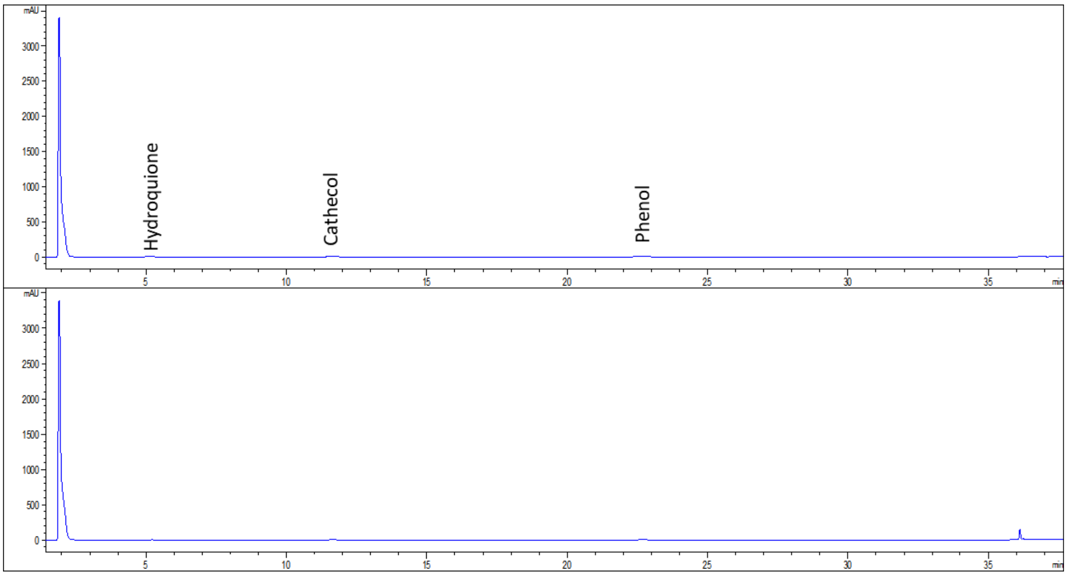


***Figure S6.*** *HPLC chromatogram of the benzene oxidation reaction in presence of $Tp^{Br3}Cu(NCMe)$ under 40 bar $O_2$ at 40 °C for 1.5 h. Top: UV detector 275 nm. Bottom: UV detector 254 nm.*

### 4. 3D-views of mono- and binuclear copper oxygen species computationally tested as potential benzene oxidants (complexes in Scheme 2)

**M1: $Cu^{II}$-superoxo (3.7)** **M2: $Cu^{II}$-hydroperoxo (15.9)** **M3: $Cu^{II}$-oxyl (38.8)**

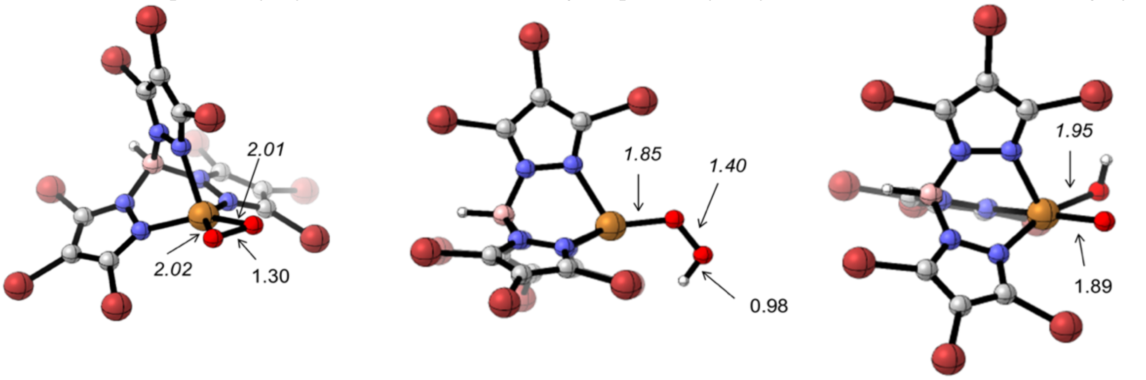


*Figure S7:* *Optimized structures of mononuclear TpBr3 Cu/O2 intermediates shown in Scheme 2. Distances in Å. In parenthesis relative Gibbs energies (in kcal mol$^{-1}$)*

**B1: $Cu^{II}$-(μ-η$^2$:η$^2$-$O_2$)-$Cu^{II}$ (-3.2)** **B2: $Cu^{II}$-(μ-O)$_2$-$Cu^{II}$ (26.4)**

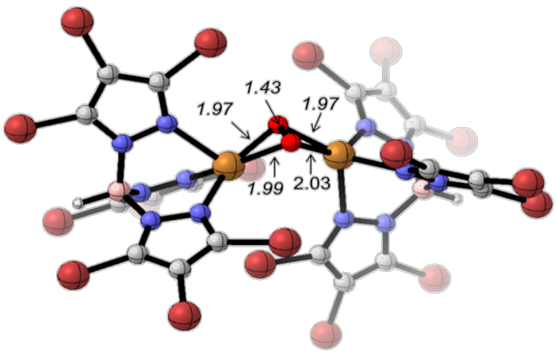


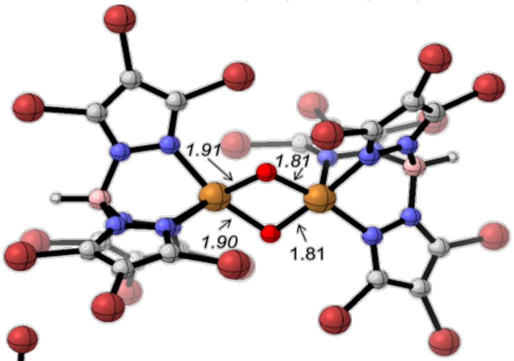


**B3: $Cu^{II}$(μ-O·)(μ-OH)$Cu^{II}$ (-7.7)**

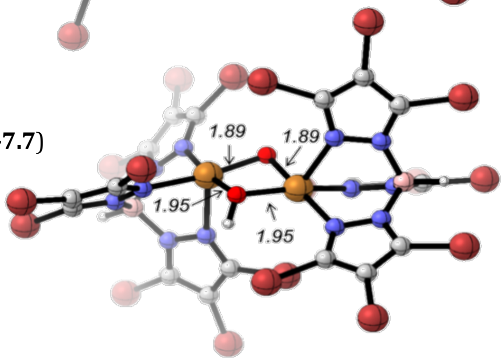


**B4: $Cu^{I}$-(μ-OOH)$_2$-$Cu^{II}$ (3.4)** **B5: $Cu^{II}$-(μ-OH)$_2$-$Cu^{II}$ (-38.5)**

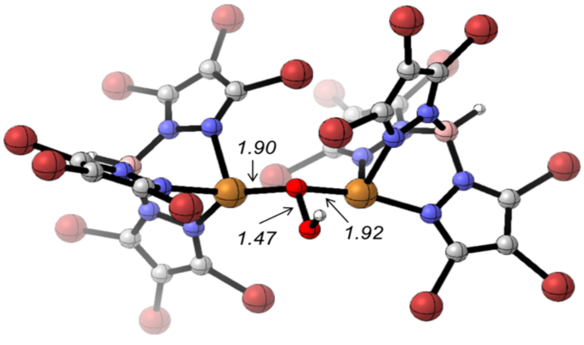


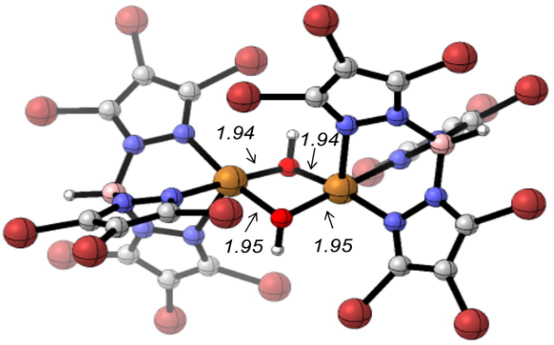


*Figure S8:* *Optimized structures of binuclear TpBr3 Cu/O2 intermediates shown in Scheme 2. Distances in Å. In parenthesis relative Gibbs energies (in kcal mol$^{-1}$)*

## 5. Gibbs energy profile for formation of active species with anionic ascorbate in the hydrogen transfer step

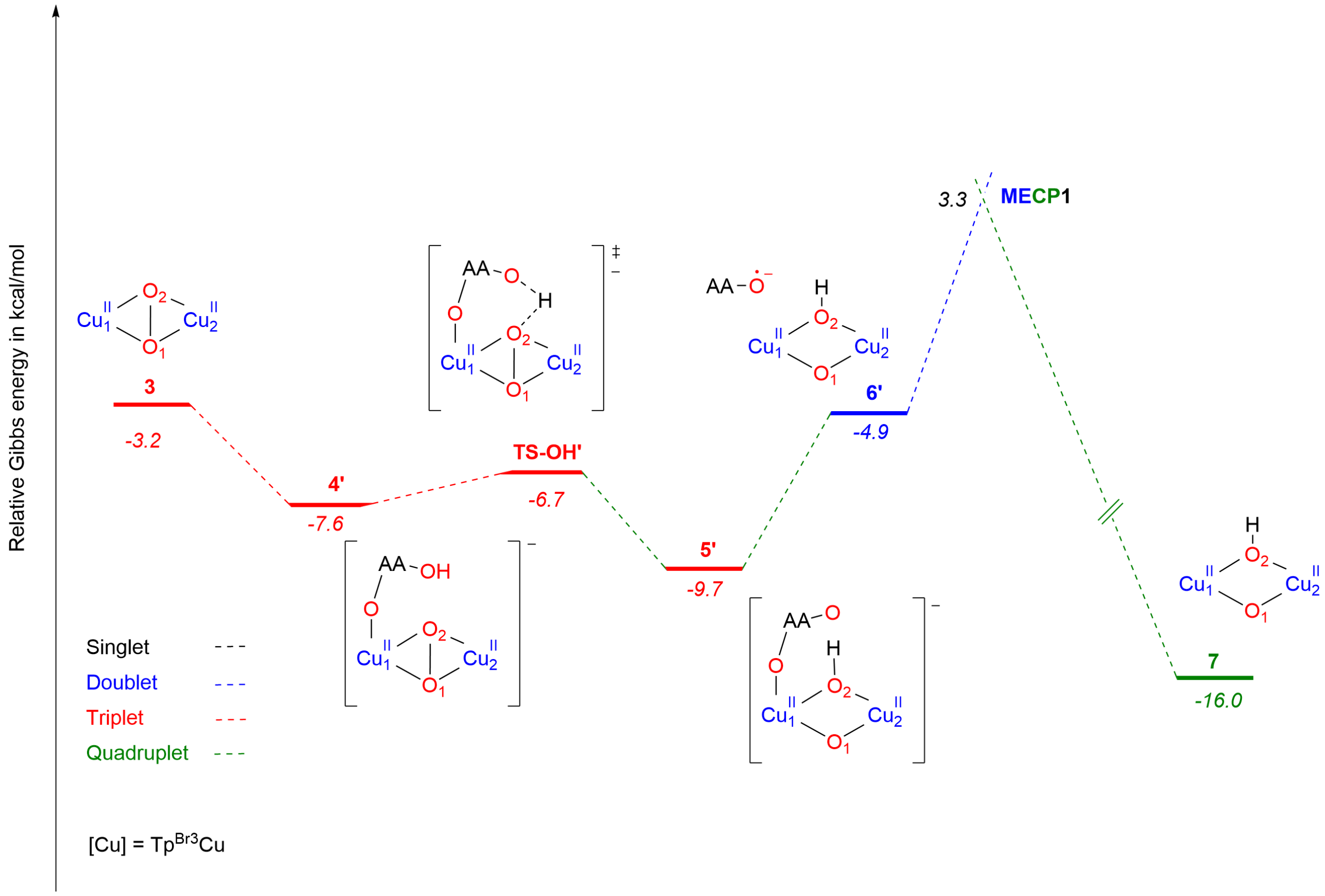


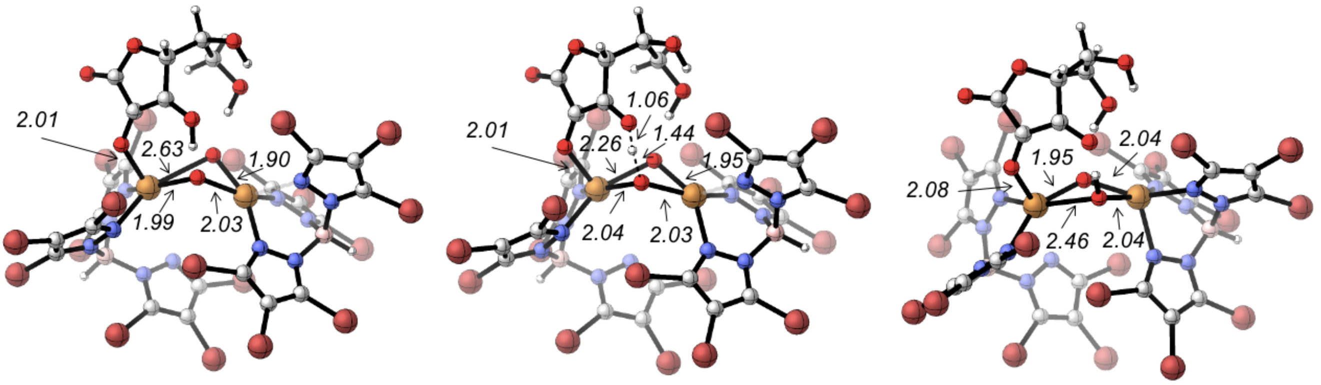


**4' (-7.6 kcal/mol)** **TS-OH' (-6.7 kcal/mol)** **5' (-9.7 kcal/mol)**

***Figure S9:*** *Gibbs energy profile for the formation of active species* **7** *considering that the Cu-OOH is formed by an anionic ascorbate (AAH⁻) . Relative Gibbs energies in acetonitrile in kcal mol-1. Bottom: Optimized structures of* **4'**, ***TS-OH'*** *and* **5'** *species.*

## 6. Dioxygen activation and HAA from benzene by mononuclear copper(I) complexes

**SM1 (15.1 kcal/mol)**
Intermediate of abstraction by **2** ($Cu^{II}$-superoxo) of a hydrogen atom from ascorbic acid

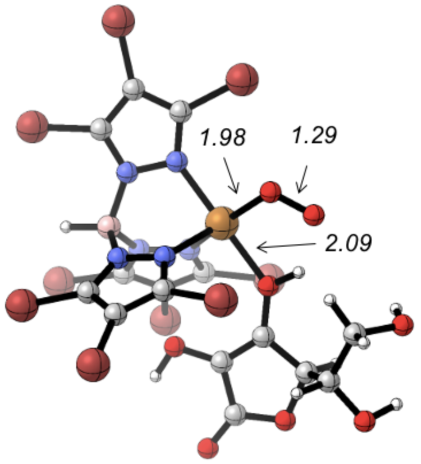


**SM2-TS (15.5 kcal/mol)**
Transition state of hydrogen abstraction from ascorbic acid by **2** ($Cu^{II}$-superoxo)

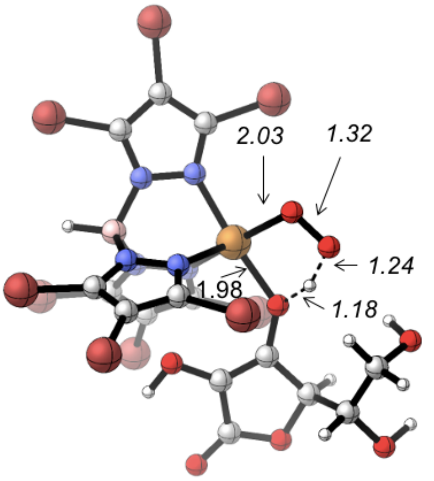


**SM3 (17.0 kcal/mol)**
$Cu^{II}$-hydroperoxo obtained from hydrogen abstraction from ascorbic acid by **2** ($Cu^{II}$-superoxo)

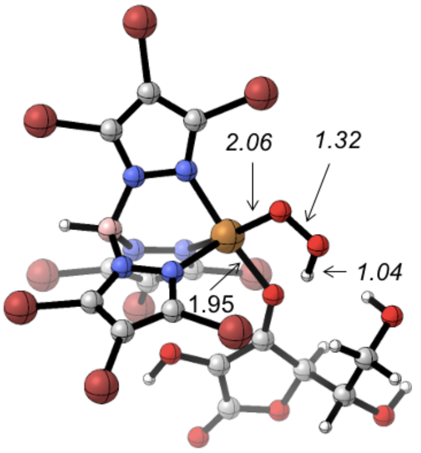


**SM1'(-3.3 kcal/mol)**
Intermediate of abstraction by **2** ($Cu^{II}$-superoxo) of a hydrogen atom from anionic ascorbic acid

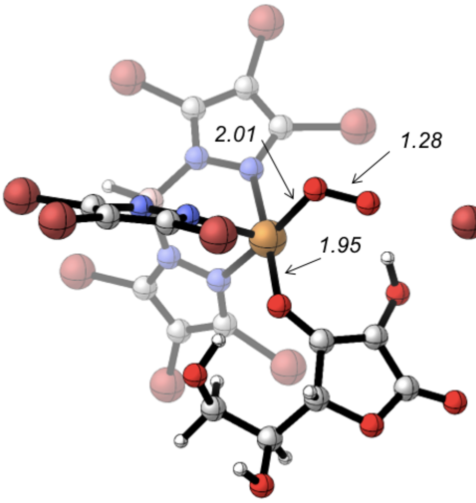


**SM2-TS'(1.0 kcal/mol)**
Transition state of hydrogen abstraction from anionic ascorbic acid by **2** ($Cu^{II}$-superoxo)

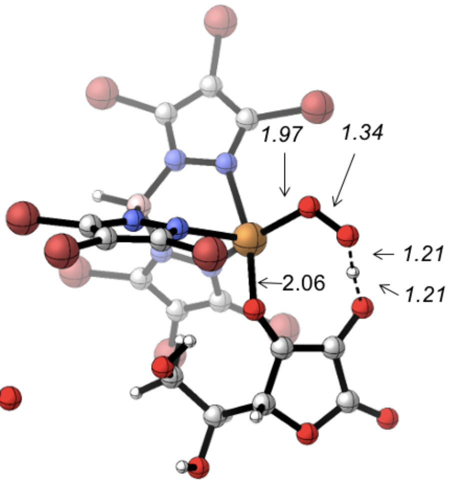


**SM3'(-0.2 kcal/mol)**
$Cu^{II}$-hydroperoxo obtained from hydrogen abstraction from anionic ascorbic acid by **2** ($Cu^{II}$-superoxo)

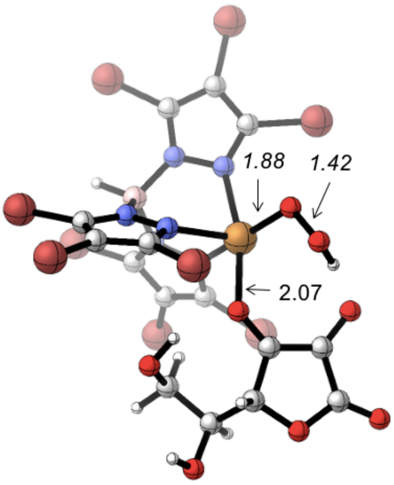


**SM4 - TS (54.5 kcal/mol)**
Transition state for HAA from benzene by $Cu^{II}$-hydroperoxo (SM2)

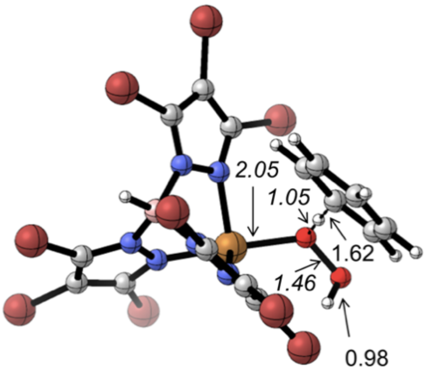


***Figure S10:*** *Optimized structures of discarded mononuclear species. Distances in Å. In parenthesis relative Gibbs energies (in kcal mol$^{-1}$)*

## 7. Dioxygen activation and HAA from benzene by binuclear copper(I) complexes

**SB1 (36.1 kcal/mol)**
Product of HAA from benzene by complex **3** $Cu^{II}$-(μ-$\eta^2$:$\eta^2$-$O_2$)-$Cu^{II}$

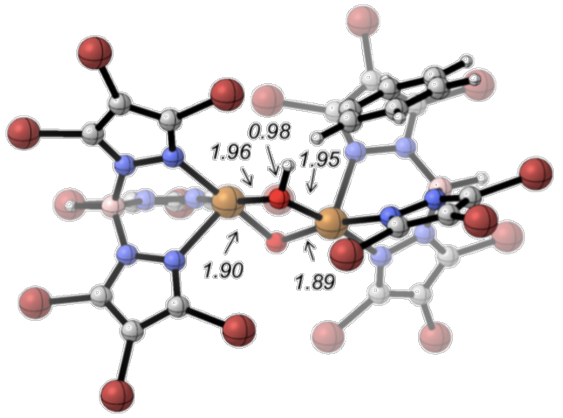


**SB2 (19.0 kcal/mol)**
$Cu^{III}$-(μ-O)$_2$-$Cu^{III}$ complex

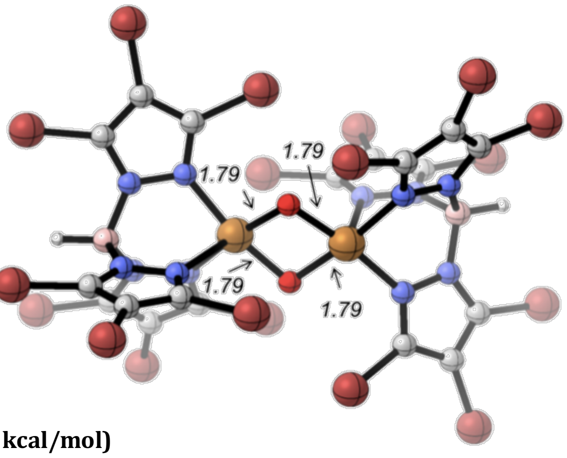


**SB3 - TS (28.8 kcal/mol)**
TS of HAA from benzene by species **6** $Cu^{I}$-(μ-OOH)-$Cu^{II}$

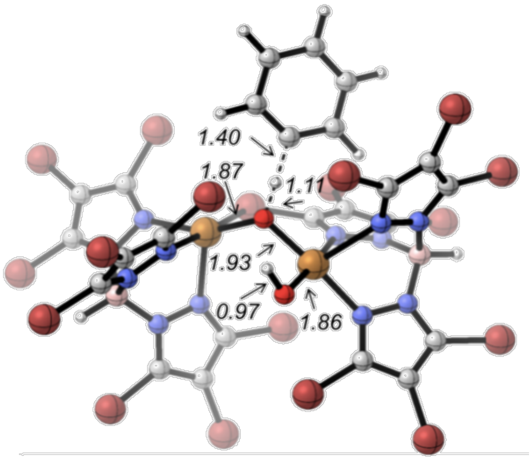


**SB4-TS (13.6 kcal/mol)**
TS for O-O bond breaking in species 6 that leads to species 7 in the doublet state

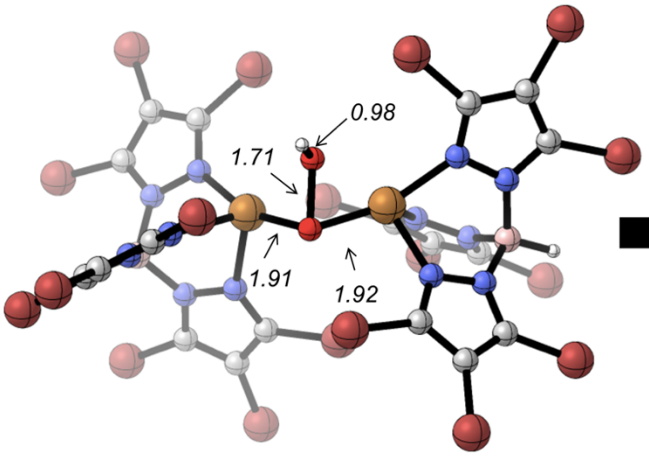


**SB5 (2.0 kcal/mol)**
Product of O-O bond breaking in species 6: 7 in the doublet state

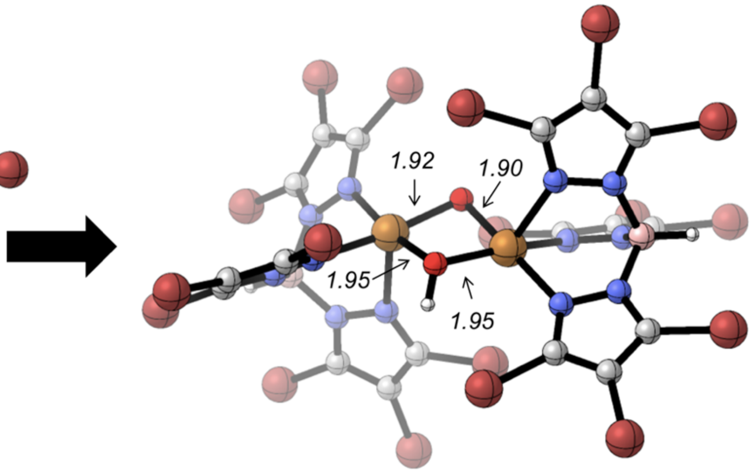


***Figure S11:*** *Optimized structures of discarded binuclear species. Distances in Å. In parenthesis relative Gibbs energies (in kcal mol*$^{-1}$*)*

## 8. HAA from $C_{sp3}$-H bonds by active species 7

**TS_CycHx (4.4 kcal/mol)**
TS of HAA from cyclohexane by species **7**

**TS_Met (12.8 kcal/mol)**
TS of HAA from methane by species **7**

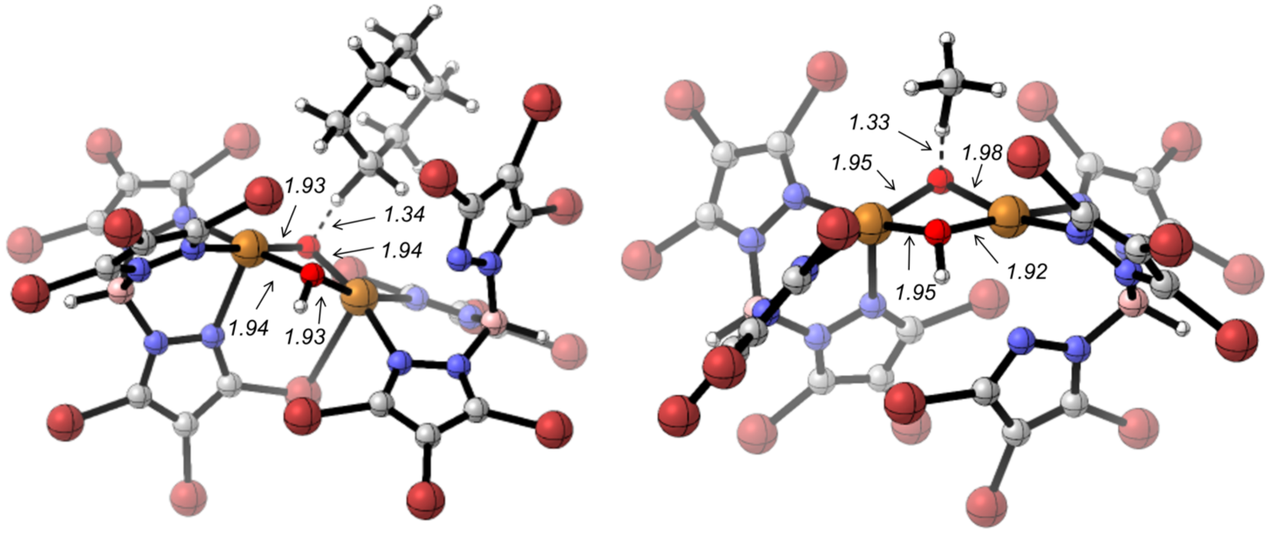


***Figure S12:*** *Optimized structures of transition states for HAA from cyclohexane (left) and methane (right) by 7. Distances in Å. In parenthesis relative Gibbs energies (in kcal mol$^{-1}$)*

## 9. DFT benchmark study of the relative stabilities of $Cu^{I}$-(μ-OOH)$_2$-$Cu^{II}$ (6, doublet) and $Cu^{II}$(μ-O·)(μ-OH)$Cu^{II}$ (7, quartet) species

***Table S1.*** *Relative energies in acetonitrile of* ***6*** *($Cu^{I}$-(μ-OOH)$_2$-$Cu^{II}$, doublet, D) and* ***7*** *($Cu^{II}$(μ-O·)(μ-OH)$Cu^{II}$, quartet, Q) intermediates with respect 2 $TpCu^{Br3}$ + $O_2$ + $AAH_2$ and Q-D energy difference (ΔE(7-6), calculated with different functionals . Energies in kcal $mol^{-1}$. All single-point calculations are done using the extendended basis set BS2 on TPSSh-D3/BS1 doublet and quadruplet optimized geometries in acetonitrile.*

| *Method* | *%HF exchange* | *6/doublet* | *7/quadruplet* | *ΔE(7-6)* |
|---|---|---|---|---|
| *PBE-D3* | 0 | -20,7 | -25,1 | -4,4 |
| *M11-L* | 0 | -23,8 | -57,9 | -34,1 |
| *TPSSh-D3* | 10 | -19,5 | -32,1 | -12,6 |
| *B3LYP-D3* | 20 | -18,0 | -29,6 | -11,6 |
| *PBE0-D3* | 25 | -9,0 | -24,6 | -15,6 |
| *M06* | 27 | -7,2 | -18,2 | -11,0 |
| *M06-D3* | 27 | -12,3 | -23,2 | -10,9 |
| *MN15* | 44 | 4,9 | 17,1 | 12,2 |
| *M06-2X-D3* | 54 | 0,9 | -14,2 | -15,1 |
| *CAM-B3LYP-D3* | 19(SR)65(LR)[a] | -12,4 | -29,3 | -16,9 |
| *wB97XD* | 22(SR)/100(LR)[a] | -5,1 | -17,7 | -12,6 |
| *M11* | 42.8(SR)/100(LR)[a] | 3,9 | -7,6 | -11,5 |

[a] *SR=short range, LR=long range*

## 10. Spin densities

***Table S2**. Mulliken spin population of all the species in the generation of the active species and benzene hydroxylation pathways*

| *Species* | *Spin State* | *Cu1* | *Cu2* | *\|ΣCu\|* | *O1* | *O2* | *ΣO* | *AA* | *CPh* |
|---|---|---|---|---|---|---|---|---|---|
| ***Generation of the active species*** | | | | | | | | | |
| ***O2*** | *T* | | | | *1* | *1* | | | |
| ***TpBr3Cu*** | *S* | *0* | *-* | *0.* | *-* | *-* | | | |
| ***2*** | *T* | *0.39* | *-* | *0.39* | *0.76* | *0.76* | *1.52* | | |
| ***3*** | *T* | *0.46* | *0.46* | *0.92* | *0.45* | *0.45* | *0.90* | | |
| ***4*** | *T* | *0.56* | *0.52* | *1.08* | *0.33* | *0.20* | *0.53* | *0.16* | |
| ***TS-OH*** | *T* | *0.56* | *0.53* | *1.09* | *0.30* | *0.16* | *0.46* | *0.20* | |
| ***5*** | *T* | *0.54* | *0.55* | *0.99* | *0.26* | *0.12* | *0.38* | *0.27* | |
| ***6*** | *D* | *0.22* | *0.43* | *0.65* | *0.18* | *0.08* | *0.26* | | |
| ***7*** | *Q* | *0.63* | *0.62* | *1.25* | *1.18* | *0.25* | *1.43* | | |
| ***Path A: Benzene hydroxylation: rebound mechanism*** | | | | | | | | | |
| ***8*** | *Q* | *0.63* | *0.63* | *1.26* | *1.17* | *0.25* | *1.42* | | |
| ***TS-CH*** | *Q* | *0.62* | *0.62* | *1.24* | *0.71* | *0.25* | *0.96* | | *0.57* |
| ***9*** | *Q* | *0.63* | *0.64* | *1.27* | *0.23* | *0.26* | *0.49* | | *0.98* |
| ***10*** | *D* | *0.63* | *0.64* | *1.27* | *0.24* | *0.26* | *0.50* | | *-0.98* |
| ***TS-CO*** | *D* | *0.63* | *-0.49* | *1.12* | *0.06* | *0.00* | *0.06* | | *0.75* |
| ***11*** | *D* | *0.48* | *0.20* | *0.68* | *0.15* | *0.03* | *0.18* | | *0.* |
| ***12*** | *T* | *0.45* | *0.23* | *0.68* | *0.11* | *0.04* | *0.15* | *0.89* | *0.* |
| ***13*** | *S* | *0.* | *0.* | *0.* | *0.* | *0.* | *0.* | *0.* | *0.* |
| *Path B:* ***Benzene hydroxylation: σ–complex based mechanism*** | | | | | | | | | |
| ***8'*** | *Q* | *0.58* | *0.63* | *1.21* | *1.19* | *0.27* | *1.46* | | |
| ***TS-CO- σ*** | *Q* | *0.57* | *0.61* | *1.17* | *0.78* | *0.26* | *1.04* | | *0.55* |
| ***σ–complex*** | *Q* | *0.61* | *0.59* | *1.20* | *0.33* | *0.25* | *0.58* | | *0.93* |
| ***σ–complex*** | *D* | *0.56* | *0.57* | *1.13* | *0.23* | *0.23* | *0.46* | | *-0.81* |
| ***σ–complex-AAH*** | *D* | *-0.53* | *0.60* | *1.13* | *0.04* | *0.01* | *0.05* | | *0.86* |
| ***TS-deprot*** | *D* | *0-02* | *0.60* | *0.02* | *0.12* | *0.14* | *0.26* | | *0.0* |
| ***PhO-AAH$_2$*** | *D* | *0.02* | *0.58* | *0* | *0.13* | *0.15* | *0.28* | | *0.0* |
| ***TS-prot*** | *D* | *0.02* | *0.58* | *0.01* | *0.15* | *0.13* | *0.28* | | *0.0* |

| | | | | | | | | | |
|---|---|---|---|---|---|---|---|---|---|
| ***PhOH-AAH*** | *D* | *0.48* | *0.22* | *0.70* | *0.03* | *0.14* | *0.14* | *1* | *0.0* |
| ***13'*** | *S* | *0.0* | *0.0* | *0.0* | *0.0* | *0.0* | *0.0* | *0.0* | *0.0* |

## 11. Absolute energies of computed species, in Hartrees

***Table S3.*** *Internal and Gibbs energies and thermal and entropic corrections (T,S correction) in acetonitrile of all the species in the generation of the active species and benzene hydroxylation pathways. For transition states the imaginary frequency is also given (in cm$^{-1}$)*

| | E/BS1 | T,S correction | G/BS1 | E/BS2 | G/BS2 | Freq |
|---|---|---|---|---|---|---|
| **TpBr3Cu** | -24035,88911 | 0,03837 | -24035,85075 | -24060,18743 | -24060,14906 | |
| **2** | -24186,23226 | 0,03991 | -24186,19235 | -24210,59080 | -24210,55089 | |
| **3** | -48222,16785 | 0,10335 | -48222,06449 | -48270,81134 | -48270,70799 | |
| **4** | -48907,01576 | 0,24387 | -48906,77190 | -48955,93144 | -48955,68757 | |
| **TS-OH** | -48907,01553 | 0,24224 | -48906,77329 | -48955,93062 | -48955,68837 | -508,4 |
| **5** | -48907,01685 | 0,24313 | -48906,77373 | -48955,93299 | -48955,68986 | |
| **6** | -48222,78250 | 0,11554 | -48222,66695 | -48271,42746 | -48271,31191 | |
| **MECP1** | -48222,77716 | 0,11825 | -48222,65892 | -48271,41704 | -48271,29879 | |
| **7** | -48222,81118 | 0,11800 | -48222,69318 | -48271,44760 | -48271,32960 | |
| **Path A: rebound mechanism** | | | | | | |
| **8** | -48455,11592 | 0,21022 | -48454,90570 | -48503,82391 | -48503,61369 | |
| **TS-CH** | -48455,08862 | 0,20406 | -48454,88456 | -48503,79676 | -48503,59271 | -1519,3 |
| **9** | -48455,10427 | 0,20938 | -48454,89489 | -48503,81284 | -48503,60346 | |
| **MECP2** | -48455,10389 | 0,21123 | -48454,89266 | -48503,81295 | -48503,60172 | |
| **10** | -48455,10412 | 0,20875 | -48454,89537 | -48503,81334 | -48503,60459 | |
| **TS-CO** | -48455,08841 | 0,20841 | -48454,88000 | -48503,79991 | -48503,59151 | -173,9 |
| **11** | -48455,18414 | 0,21598 | -48454,96816 | -48503,89663 | -48503,68065 | |
| **12** | -49139,41057 | 0,34068 | -49139,06989 | -49188,39610 | -49188,05541 | |
| **MECP3** | -49139,40290 | 0,33614 | -49139,06676 | -49188,39052 | -49188,05438 | |
| **13** | -49139,42358 | 0,33762 | -49139,08596 | -49188,41286 | -49188,07524 | |
| **14** | -48831,89184 | 0,23900 | -48831,65283 | -48880,77767 | -48880,53866 | |
| **Path B: σ-complex based mechanism** | | | | | | |
| **8'** | -48455,11581 | 0,21369 | -48454,90212 | -48503,82396 | -48503,61027 | |
| **TS-CO-σ** | -48455,09553 | 0,21359 | -48454,88194 | -48503,80355 | -48503,58996 | -383.2 |
| **σ–complex** | -48455,11303 | 0,21737 | -48454,89566 | -48503,82258 | -48503,60521 | |
| **σ–complex** | -48455,11465 | 0,21761 | -48454,89705 | -48503,82459 | -48503,60698 | |
| **σ–complex-AAH** | -49139,49259 | 0,34076 | -49139,15183 | -49188,48440 | -49188,14364 | |
| **TS-deprot** | -49139,48449 | 0,33402 | -49139,15047 | -49188,47838 | -49188,14436 | -9.9 |
| **PhO-AAH$_2$** | -49139,51462 | 0,33494 | -49139,17969 | -49188,50994 | -49188,17501 | |
| **TS-prot** | -49139,51181 | 0,33467 | -49139,17714 | -49188,50743 | -49188,17276 | -51.8 |
| **PhOH-AAH** | -49139,55855 | 0,33780 | -49139,22075 | -49188,55431 | -49188,21651 | |
| **13'** | -48455,34810 | 0,21166 | -48455,13644 | -48504,07400 | -48503,86235 | |

***Table S4.*** *Internal and Gibbs energies and thermal and entropic corrections (T,S correction) in acetonitrile for the formation of active species with anionic ascorbic acid in Figure S6*

| | E/BS1 | T,S correction | G/BS1 | E/BS2 | G/BS2 | Freq |
|---|---|---|---|---|---|---|
| **4'** | -48906,56433 | 0,22779 | -48906,33654 | -48955,48114 | -48955,25335 | |
| **TS-OH'** | -48906,56352 | 0,22804 | -48906,33548 | -48955,47990 | -48955,25186 | -118.8 |
| **5'** | -48906,56840 | 0,22947 | -48906,33893 | -48955,48613 | -48955,25666 | |

***Table S5.*** *Internal and Gibbs energies and thermal and entropic corrections (T,S correction) in acetonitrile of all the mononuclear (**M**) and binuclear (**B**) species in Scheme 2 and Figures S7 and S8*

| | E/BS1 | T,S correction | G/BS1 | E/BS2 | G/BS2 |
|---|---|---|---|---|---|
| **M1** | -24186,23226 | 0,03991 | -24186,19235 | -24210,59080 | -24210,55089 |
| **M2** | -24186,83947 | 0,05225 | -24186,78722 | -24211,19824 | -24211,14599 |
| **M3** | -24186,80545 | 0,05033 | -24186,75513 | -24211,15983 | -24211,10950 |
| **B1** | -48222,16785 | 0,10335 | -48222,06449 | -48270,81134 | -48270,70799 |
| **B2** | -48222,12939 | 0,10517 | -48222,02422 | -48270,76608 | -48270,66091 |
| **B3** | -48222,81118 | 0,11800 | -48222,69318 | -48271,44760 | -48271,32960 |
| **B4** | -48222,78250 | 0,11554 | -48222,66695 | -48271,42746 | -48271,31191 |
| **B5** | -48223,48300 | 0,12779 | -48223,35520 | -48272,12097 | -48271,99318 |

***Table S6**. Internal and Gibbs energies and thermal and entropic corrections (T,S correction) in acetonitrile of the species in Figures S9, S10 and S11. For transition states the imaginary frequency is also given (in cm$^{-1}$)*

| | ***E/BS1*** | ***T,S correction*** | ***G/BS1*** | ***E/BS2*** | ***G/BS2*** | ***Freq*** |
|---|---|---|---|---|---|---|
| ***SM1*** | -24871,06484 | 0,17676 | -24870,88807 | -24895,69944 | -24895,52268 | |
| ***SM2-TS*** | -24871,06259 | 0,17422 | -24870,88836 | -24895,69630 | -24895,52208 | -870.6 |
| ***SM3*** | -24871,06499 | 0,17848 | -24870,88651 | -24895,69811 | -24895,51963 | |
| ***SM1'*** | -24870,62257 | 0,162293 | -24870,46028 | -24895,26267 | -24895,10038 | |
| ***SM2-TS'*** | -24870,60942 | 0,154958 | -24870,45446 | -24895,24863 | -24895,09367 | -1450.3 |
| ***SM3'*** | -24870,61846 | 0,162016 | -24870,45644 | -24895,25756 | -24895,09555 | |
| ***SM4-TS*** | -24419,07412 | 0,13881 | -24418,93531 | -24443,50911 | -24443,37030 | -310,5 |
| ***SB1*** | -48454,43189 | 0,19818 | -48454,23370 | -48503,12943 | -48502,93125 | |
| ***SB2*** | -48222,14236 | 0,10749 | -48222,03487 | -48270,78010 | -48270,67261 | |
| ***SB3 - TS*** | -48455,05285 | 0,20235 | -48454,85050 | -48503,76273 | -48503,56039 | -909,1 |
| ***SB4 - TS*** | -48222,76382 | 0,11206 | -48222,65176 | -48271,40776 | -48271,29570 | -682,6 |
| ***SB5*** | -48222,80572 | 0,11765 | -48222,68807 | -48271,43822 | -48271,32058 | |
| ***TS-CyHx*** | -48458,74883 | 0,27510 | -48458,47373 | -48507,45023 | -48507,17513 | -575,2 |
| ***TS-Met*** | -48263,32522 | 0,15803 | -48263,16719 | -48311,97300 | -48311,81497 | -1612,7 |
